# Co-expression of auxiliary genes enhances the activity of a heterologous O_2_-tolerant hydrogenase in the cyanobacterium *Synechocystis* sp. PCC 6803

**DOI:** 10.1186/s13068-025-02634-5

**Published:** 2025-03-28

**Authors:** Sara Lupacchini, Ron Stauder, Franz Opel, Stephan Klähn, Andreas Schmid, Bruno Bühler, Jörg Toepel

**Affiliations:** 1 Department of Microbial Biotechnology, Helmholtz Centre for Environmental Research-UFZ, Permoserstrasse 15, 04318 Leipzig, Germany; 2 Department of Solar Materials Biotechnology, Helmholtz Centre for Environmental Research-UFZ, Permoserstrasse 15, 04318 Leipzig, Germany

**Keywords:** Oxygen-tolerant hydrogenase, Oxygenic photosynthesis, Cyanobacteria, Hydrogenase maturation

## Abstract

Cyanobacteria bear great biotechnological potential as photosynthetic cell factories. In particular, hydrogenases are promising with respect to light-driven H_2_ production as well as H_2_-driven redox biocatalysis. Their utilization relies on effective strain design as well as a balanced synthesis and maturation of heterologous enzymes. In a previous study, the soluble O_2_-tolerant hydrogenase complex from *Cupriavidus necator *(*Cn*SH) could be introduced into the model cyanobacterium *Synechocystis* sp. PCC 6803. Due to its O_2_-tolerance, it was indeed active under photoautotrophic growth conditions. However, the specific activity was rather low indicating that further engineering is required, for which we followed a two-step approach. First, we optimized the *Cn*SH multigene expression in *Synechocystis* by applying different regulatory elements. Although corresponding protein levels and specific *Cn*SH activity increased, the apparent rise in enzyme levels did not fully translate into activity increase. Second, the entire set of *hyp* genes, encoding *Cn*SH maturases, was co-expressed in *Synechocystis* to investigate, if *Cn*SH maturation was limiting. Indeed, the native *Cn*SH maturation apparatus promoted functional *Cn*SH synthesis, enabling a threefold higher H_2_ oxidation activity compared to the parental strain. Our results suggest that a fine balance between heterologous hydrogenase and maturase expression is required to ensure high specific activity over an extended time period.

**Supplementary Information:**

The online version contains supplementary material available at 10.1186/s13068-025-02634-5.

## Introduction

H_2_ is considered a key element of future cyclic economies and is of major interest within the field of renewable energy [55]. Despite its potential for a decarbonized economy, 96% of H_2_ production still relies on fossil resource usage. In addition to technical issues regarding storage and H_2_ conversion, the big challenge is to develop sustainable ways for H_2_ production [23]. Nature comes in by showing diverse processes for microbial H_2_ production [22]. Many microorganisms can produce H_2_ via dark and photo-fermentation [59], and oxygenic photosynthesis, deriving electrons from water oxidation, in principle can be coupled with H_2_ production [9, 10]. Most prominent is the application of either eukaryotic microalgae or cyanobacteria for such light-driven H_2_ production (photo-H_2_). In green algae, H_2_ formation relies on [FeFe] hydrogenases, which show high turnover rates of up to 104 s^−1^. They are, however, produced and active only under micro- or anaerobic conditions and are rapidly disintegrated in the presence of molecular oxygen [51]. In contrast, cyanobacteria typically feature bidirectional [NiFe] hydrogenases, which are not disintegrated in the presence of O_2_, but reversibly inhibited. The unicellular model cyanobacterium *Synechocystis* sp. PCC 6803 (hereafter *Synechocystis*) harbors a pentameric enzyme (*Syn*SH) composed of a hydrogenase module (HoxYH) and a diaphorase module (HoxEFU) [63]. *Syn*SH is associated to the thylakoid membrane by means of the HoxE subunit [7], which facilitates electron transfer from the photosynthetic electron transport chain to the diaphorase module HoxEFU via reduced flavodoxins and ferredoxins [20]. *Syn*SH is considered to work as an electron valve to compensate for transiently missing electron sinks such as the Calvin-Bassham-Benson (CBB) cycle and O_2_ upon sudden switches from dark to light [1, 44]. Recently, an involvement of *Syn*SH in electron balancing under oxic conditions has been proposed, indicating a multi-functional role of this enzyme in cyanobacteria [6].

The main limiting factors for applying cyanobacterial hydrogenases for photo-H_2_ production are its O_2_-sensitivity, H_2_ re-oxidation when C- and N-assimilatory pathways as native electron acceptors become active, and the competition with these for photosynthetically derived electrons [1]. During the past two decades, progress has been made to overcome these challenges [34]. Recently, photosynthetic electron flow towards H_2_ formation instead of nitrate, CO_2_, and/or O_2_ reduction has been targeted via metabolic engineering [2, 11, 21, 31, 45], and a direct coupling of HoxYH of *Synechocystis* to photosystem I (PSI) has been established [1, 32]. The latter approach resulted in reduced competition with the downstream metabolic pathways and avoided H_2_ uptake activity. Photo-H_2_ production has also been facilitated via enzymatic O_2_-removal, though O_2_-sensitivity and electron transfer efficiency remain challenges to be addressed [51].

One possible approach to circumvent the O_2_ problem is the utilization of natural O_2_-tolerant hydrogenases. In this respect, heterologous expression of functional [NiFe] hydrogenases tolerating up to 1–3% of O_2_ has been achieved in *Synechococcus elongatus* [67]. Moreover, the “Knallgas” bacterium *Cupriavidus necator* (hereafter *C. necator*) features a soluble [NiFe] hydrogenase (*Cn*SH) even retaining full activity at 20% O_2_ [26, 37]. *Cn*SH has successfully been introduced in heterotrophic hosts [18, 36, 52, 57] and, recently, also in *Synechocystis* [42]. In *Synechocystis*, *Cn*SH was continuously active during oxygenic photosynthesis, oxidizing H_2_ independently of the O_2_ concentration. The results revealed a tight interconnection of *Cn*SH with cyanobacterial metabolism. The engineered strain *Syn_Cn*SH^+^ was able to use H_2_-derived electrons to fix CO_2_ and fuel growth even in the absence of water oxidation activity [42]. However, due to the strict dependency of *Cn*SH on NADH as electron donor, H_2_ formation was achieved only in the presence of glucose effecting an elevated cytosolic NADH/NAD^+^ ratio. For application of *Cn*SH for photo-H_2_ production, it is, therefore, required to either provide electrons in the form of NADH, change its electron donor specificity, or couple the hydrogenase module directly to PSI.

Moreover, the specific *Cn*SH activity exhibited by *Syn_Cn*SH^+^ was up to two orders of magnitude lower than that in its native host [42, 52, 53]. This can be explained by lower enzyme abundance and/or inefficient hydrogenase maturation. Interestingly, a parallel introduction of the *C. necator* maturation apparatus was not in all cases required to establish a functional *Cn*SH in heterotrophic hosts. Key differences among these studies include the genetic background of the host strains, growth conditions applied, and expression systems used for the multi-gene operon. To achieve *Cn*SH activity in *Synechocystis*, the expression of auxiliary genes for *Cn*SH maturation was not required, except for *hox*W encoding a HoxH-specific endopeptidase [42]. Obviously, hydrogenase maturation factors of *Synechocystis,* encoded by the 6 accessory genes *hyp*A1, *hyp*B1, *hyp*C, *hyp*D, *hyp*E, and *hyp*F [25], to some extent also enable functional *Cn*SH assembly in *Synechocystis* under aerobic conditions. However, the specific *Cn*SH activity in cell-free extract of *Syn_Cn*SH^+^ was roughly 200 times lower than those reported for *C. necator* or recombinant *E. coli* or *P. putida* with *Cn*SH genes and corresponding *hyp* genes co-expressed.

In this study, we characterized limitations for *Cn*SH specific activities in *Synechocystis* [42] and increased the specific activity three-fold by fine-tuning *Cn*SH multigene expression and co-expression *C. necator hyp* genes.

## Materials and methods

### Cloning strategy and strain engineering

Shuttle vectors were built as part of a modular cloning strategy based on the established Golden Gate cloning system [12, 66]. The resulting system is comparable to the previously published CyanoGate [62], with the possibility to easily create a library of different genetic elements and assemble them in the desired way. The usage of type IIS restriction enzymes, like *BpiI* and *BsaI*, allows the simultaneous assembly of multiple DNA fragments in correct orientations. Compared to the CyanoGate system, the order of restriction enzymes was switched in this work and combined with different restriction site overhangs. A schematic view of the procedure from the amplification of each basal genetic element until the level 2 assembly is represented in Fig. S1. As in the CyanoGate system, the genetic elements of interest initially were cloned in the so-called level 0 entry vectors via *BsaI* restriction site overlaps. In the second step, each genetic element was cloned into level 1 “positioning level” vectors using *BpiI*. Level 1 vectors were designed to contain *BsaI* restriction sites up- and downstream of *BpiI* restriction sites. We generated level 1 vectors for 7 positions in total. After *BsaI* digestion, each positioning vector featured specific overhangs matching only with the overhangs of the follow up positioning vector after its digestion. In the last step, up to 7 genetic elements from level 1 vectors were assembled into final level 2 expression vectors, which then were introduced into *Syn*_∆hox. We used pGGC 212 for genome integration and pGGC 209 as a replicative vector based on the pSEVA351 backbone [43]. Modular assembly protocols for level 0, 1, and 2 generation are summarized in Table S1. Primer, promoter, RBS, terminator, and end-linker sequences are summarized in Table S2 and the constructed vectors in Table S3. The level 2 vector pGGC 212 was introduced via natural transformation into *Syn*_∆*hox*, where the constructed *Cn*_*hox* operon replaced the kanamycin cassette as previously described [42], giving strain *Syn_P*_*nrsB*_*Cn*SHg. Electro-competent *Syn*_∆hox cells we transformed with the replicative pGGC 209 vector according to a standard protocol [5], giving the strain *Syn_P*_*nrsB*_*Cn*SHp.

The modular assembly strategy also was used to generate replicative plasmids harboring the entire *hyp* operon under the control of 3 different promotors (*P*_*nrsB*_*, P*_*rha*_*, P*_*psbA2*_). Additionally, plasmids just carrying the *hypX* gene of *C. necator* under control of the *P*_*nrsB*_ or the *P*_*rha*_ promotor were constructed. The resulting level 2 expression vectors (pGGC 271, 272, 273, 243, and 244), all based on the pSEVA351 backbone [43], were transformed via electroporation into *Syn_P*_*nrsB*_*Cn*SHg and are summarized in Table S4.

### Growth conditions

Cyanobacterial cells were incubated in baffled Erlenmeyer flasks in yBG11 medium with 10 mM HEPES buffer (pH 7.2) [54] in growth chambers (Minitron LED Option HT, Infors, Bottmingen, Switzerland) at 30 °C under continuous illumination with 50 μmol photons m^−2^ s^−1^, continuous shaking at 150 rpm (amplitude 2.5 cm), and a CO_2_-enriched (2% [v/v]) atmosphere. Humidity was kept constant at 75%. Optionally, nickel sulfate and ferric ammonium citrate were supplemented for induction and support of *Cn*SH maturation, respectively. While the concentration of ferric ammonium citrate was 17 µM for all applications [49], the nickel sulfate concentration was varied between 2.5 and 10 µM. When specified, l-rhamnose was added to the culture 48 h before cell harvesting for *Cn_hyp* induction applying final concentrations of 0.1 or 2 mM.

### RNA isolation and analysis of transcript abundance

For RNA isolation, cells of *Syn_P*_*nrsB*_*Cn*SHg containing p*P*_*nrsB*_*Cn*Hyp, p*P*_*rhaBAD*_*Cn*Hyp, or p*P*_*psbA2*_*Cn*Hyp were grown until an OD_750_ ~ 0.8. Then, cultures were supplemented with 10 μM NiSO_4_ to induce Cn_*hox* expression and the *Cn_hyp* operon in cells containing p*P*_*nrsB*_*Cn*Hyp*.* Strains containing p*P*_*rhaBAD*_*Cn*Hyp were additionally supplied with 0.1 or 2 mM l-rhamnose. Immediately before (time 0 h) and 24 h after induction, cells were harvested by centrifugation (5000*g*, 10 min, 4 °C). RNA isolation was performed as described previously [4]. Then, 550 ng RNA of each sample was subjected to DNase I digestion (Thermo Fisher Scientific, Waltham, USA) according to the manufacturer’s instructions. cDNA synthesis was then performed using the High-Capacity cDNA Reverse Transcription kit (Thermo Fisher Scientific) following the manufacturer’s instructions. The amplified cDNA was diluted 1:10 and applied for qRT-PCR using the Power SYBR Green Mastermix (Thermo Fisher Scientific) and a StepOnePlus™ Real-Time PCR System (Thermo Fisher Scientific) according to the manufacturer’s instructions. Sequences of primer pairs for the amplification of specific regions within *rnpB* (RS 297/298) serving as reference gene [50], *hypA* (RS 295/296), and *hypX* (RS 308/309) are given in Table S2. Relative abundances normalized to *rnpB* were calculated (ΔΔCt method) [40]. Further, relative transcript levels of target genes in induced strains were normalized to those in non-induced control strains.

### Cell disruption and immunodetection

*Syn*_∆hox, *Syn_Cn*SH^+^, *Syn_P*_*nrsB*_*Cn*SHp, and *Syn_P*_*nrsB*_*Cn*SHg cultures (OD_750_ 3–4) were harvested 24 h after the addition of ferric ammonium citrate and nickel sulfate. The procedure to obtain crude cell extract for protein analysis and in vitro assays was as described previously [42]. For Western blot analysis, soluble fractions containing 20–30 µg total protein were mixed with the same volume of 2 × SDS loading dye buffer (121.14 g L^−1^ Tris HCl, 40 g L^−1^ SDS, 30.8 g L^−1^ DTT, 0.5 g L^−1^ bromophenol blue, 200 g L^−1^ glycerol) and heated to 99 °C for 10 min for complete protein denaturation. Proteins were separated by electrophoresis [35] on non-denaturing polyacrylamide gradient gels (4–15% Mini-PROTEAN TGX Precast Gels, BioRad, Hercules, USA), using an SDS running buffer (3.03 g L^−1^ Tris HCl, 1 g L^−1^ SDS, 18.77 g L^−1^ glycine, pH 8.3), for about 80 min at 120 V. The Protein Ladder SM26616 (Thermo Fisher Scientific) was loaded next to protein samples. For blotting, standard procedures were followed [58]. Specifically, 6 × Whatman filter papers, 1 × 0.45 μm pore size nitrocellulose membrane (GVS), and the SDS gel were stacked and equilibrated for 5 min in the blotting buffer (3 g L^−1^ Tris HCl, 14.4 g L^−1^ glycine, 200 mL L^−1^ MeOH) before blotting for 30 min (0.8 mA cm^−2^, Biometra P25, Analytik Jena, Jena, Germany). The blotted membrane was then blocked in TTBS buffer (0.05 M Tris HCl, 0.15 M NaCl, pH 7.4, 0.05% (v/v) Tween 20) containing 5% (w/v) BSA on a rocking table for 1 h. Then, the antibody against HoxH (0.35 g L^−1^, Eurogentec, Seraing, Belgium) was added at a dilution of 1:20.000, and the membrane was hybridized overnight at 4 °C. Afterwards, the membrane was carefully washed with TTBS and incubated for 1 h in TTBS + 3% (w/v) BSA with Goat anti-Rabbit IgG HRP-conjugate, diluted 1:500 (10 µg mL^−1^, Invitrogen, Carlsbad, USA). Finally, after washing 5 × with TTBS, the membrane was supplied with substrate solution WesternBright ECL (Advansta, San Jose, USA) and subjected to chemiluminescence detection using a Fusion FX7 EDGE V0.7 imaging system (VILBER, Eberhardzell, Germany) following the manufacturer’s instructions. As loading control for Western blot analysis, 10 µg of total soluble protein from the same samples were run on SDS-PAGE according to Laemmli [35]. The gel was stained with Coomassie Brillant Blue R-250.

### In-gel activity staining and determination of in vitro H_2_-oxidation activity

In-gel activity staining was performed as described previously [42]. In short, soluble protein fractions were diluted in loading dye solution (50 mM KPi buffer, pH 7.0, 50 g L^−1^ glycerol, 2.5 g L^−1^ bromophenol blue) and separated on non-denaturing polyacrylamide gradient gels (4–15% Mini-PROTEAN TGX Precast Gels, BioRad) at 4 °C. The gel was then incubated for 30 min at 30 °C in an airtight 120 mL bottle containing 100 mL H_2_-saturated 50 mM Tris HCl buffer, pH 8.0. Then, 800 µM NAD^+^ and 60 µM NBT were added followed by dark incubation for 30 min at 30 °C. NBT reduction by NADH emerging from hydrogenase-catalyzed H_2_ oxidation results in a dark blue precipitation visualizing the location of the active hydrogenase complex on the gel.

For the in-vitro H_2_ oxidation activity determination, NADH formation by soluble cell extracts was followed using a Cary Bio 300 UV‐visible spectrophotometer (Varian, Palo Alto, USA) as described before [42], with the following specifics: H_2_ saturated buffer was supplemented with 1 mM NAD^+^ and 1 µM FMN, while DTT was left out, because it is not helpful for maintaining the *Cn*SH fully active when the assay is performed with soluble cell extract [38]. Higher activities for *Syn_Cn*SH^+^ compared to published data rely mainly on the use of soluble cell extracts obtained directly from growing cultures instead of frozen cell pellets stored at −20 °C.

### In vivo H_2_ consumption measured via gas chromatography (GC)

*Synechocystis* cultures were grown photoautotrophically with 2% CO_2_ as described above until an OD_750_ of 2.5–5. Samples (5 mL) of each strain were transferred into 10 mL glass vials (10 mL Crimp Top HS Vial, Thermo Fisher Scientific) closed with gas-tight caps (ND20 magnetic crimp cap, Aluminium, 10 mm center hole, septa molded butyl, 3.0 mm, 55° shore A, Th. Geyer, Renningen, Germany). The headspace of the sealed vials was flushed for 1 min with a gas mixture of 20% H_2_, 10% CO_2_, and 70% N_2_ using a gas flow mixing station (PCU-10 Display and Control Device, Vögtlin, Muttenz, Switzerland). The closed vials were incubated at 30 °C, 50 µmol photons m^−2^ s^−1^ and 150 rpm. H_2_ concentrations were measured every 2 h during the incubation time. Gas analysis was conducted on a TRACE 1310 gas chromatograph (Thermo Fisher Scientific) equipped with a TracePLOT TG-BOND Sieve 5A column (length: 30 m; inside diameter: 0.32 mm; film thickness: 0.30 μm, Thermo Fisher Scientific). Other settings: Thermal Conductivity Detector (TCD): 100 °C and Oven: 50 °C. A sample volume of 100 μL was injected using the TriPlusRSH automated injection. The isothermic carrier gas (argon) flow rate was set to 2 mL min^−1^. The total run time was 2.4 min. H_2_ and O_2_ were quantified using calibration curves of both gases, determined with defined gas mixtures (Air Products, Allentown, USA). Specific activities were calculated in U (1 U corresponds to the consumption of 1 μmol H_2_ min^−1^) per g of cell dry weight (CDW). One mL of cell suspension was adjusted to a cell dry weight (CDW) of 1 g_CDW_ L^−1^ using a correlation factor of 0.225 g_CDW_ L^−1^ for OD_750_ = 1 as determined previously [27].

## Results

### Design of an optimized and controllable *hox* gene cluster for improved *Cn*SH production in *Synechocystis*

In our previous study, the *hoxFUYHW* operon from *C. necator* that encodes all components of *Cn*SH has successfully been introduced into the *Synechocystis* chromosome. Gene expression was mediated by the light-dependent *psbA2* promoter. However, even though enzyme activity could be detected under photoautotrophic growth conditions, i.e., during water oxidation and O_2_ release, the achieved specific hydrogenase activity was rather low (data not shown). Accordingly, we aimed at increasing *Cn*SH production by modulating and improving *hoxFUYHW* expression in *Synechocystis.* For this purpose, we designed a novel construct using different genetic elements such as alternative promoters, effective ribosome binding sites (RBS), as well as transcriptional terminators.

**Fig. 1:**
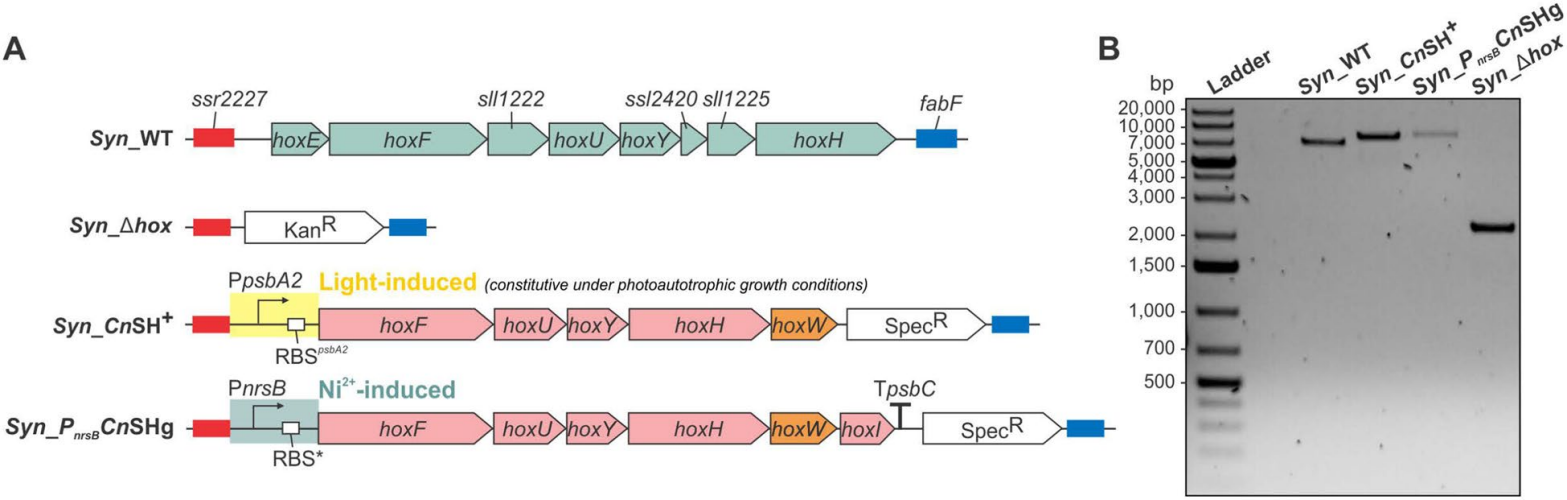
Schematic overview and confirmation of the *hox* region in parent and constructed *Synechocystis* strains. **A** In *Syn_Cn*SH^+^, *Cn*SH is encoded by a pentacistronic operon under the control of the light inducible *psbA2* promoter [42]. The cassette was integrated into the genomic region harboring the native hydrogenase genes in wildtype *Synechocystis* (*Syn*_WT). As a negative control and parent strain for strain construction, a Δ*hox* strain with the native *hox* genes replaced by a kanamycin resistance cassette (Syn_ Δ*hox*) was used. The constructed *Cn*SH expression system *P*_*nrsB*_*Cn*SH contains the complete *Cn*SH operon *(hoxFUYHWI*) enclosed by the nickel-inducible *nrsB* promoter, the synthetic RBS*, and the *psbC* terminator (T_*psbC*_), all from *Synechocystis* and was assembled on the pBluescript II SK(+) vector for chromosomal integration to generate *Syn*_*P*_*nrsB*_*Cn*SHg. Blue and red rectangles: homologous regions,Kan^R^: kanamycin resistance cassette; Spec^R^: spectinomycin resistance cassette. **B** Colony-PCR confirming the correct genetic background of the strains represented in panel A, with primers indicated in Table S2. Ladder: GeneRuler 1 kb Plus DNA Ladder, 75–20,000 bp (Thermo Fisher Scientific)

Several promoters of *Synechocystis* have been characterized for biotechnological applications [3, 13, 39, 46, 71]. Among the inducible systems, we chose the promoter of the *nrsB* gene (*P*_*nrsB*_) that has previously been used for heterologous gene expression in *Synechocystis*, showed low basal activity, and enabled strong induction by micromolar concentrations of nickel ions [48, 61]. Moreover, we opted for the synthetic RBS*, which is widely used in *Synechocystis* for heterologous gene expression as it provides high rates of translation [24]. Further, we made use of the Rho-independent transcriptional terminator T_*psbC*_ to stabilize gene expression by preventing interference with downstream genes, especially upon chromosomal integration [17, 33, 39]. Besides changing these regulatory elements compared to *Syn*_*Cn*SH^+^, the *hoxI* gene was included in the hexacistronic operon *hoxFUYHWI* resembling the gene organization in the native host. The final construct was assembled either in an episomal plasmid or integrated into the chromosome to compare plasmid- and genome-based expression. Specifically, the application of the RSF1010-based plasmid was chosen to achieve possibly higher expression levels due to a high gene copy number. Chromosomal integration was intended to establish long-term stable expression, minimizing loss of function across generations, eliminating the continuous need for selection pressure [30], and being aware that *Synechocystis* contains multiple genome copies, which can benefit genome-based expression [69]. A modified version of the CyanoGate system [62] was developed to generate various genetic configurations, which can be transferred in replicative pSEVA plasmids or in those that enable chromosomal integration in *Synechocystis* (see methods section and supplemental material for details). This finally resulted in the strains *Syn*_*P*_*nrsB*_*Cn*SHg, with the construct P*nrsB*::RBS*::*hoxFUYHWI*::T*psbC* replacing a kanamycin resistance cassette previously used to knock out the native *hox* genes of *Synechocystis* (Fig. 1A), and *Syn*_*P*_*nrsB*_*Cn*SHp, containing the same construct on a replicative vector. The maternal strain *Syn*_Δ*hox* [42] served as a negative control during strain evaluation. Colony PCR confirmed the correct genetic configurations (Figs. 1B and S2).

### Recombinant *Synechocystis* strains with tunable *hox* gene expression show improved specific CnSH activity

To confirm heterologous *hox* gene expression in *Syn_P*_*nrsB*_*Cn*SHg and *Syn*_*P*_*nrsB*_*Cn*SHp, *Cn*SH production and activity were systematically analyzed and compared to the previously generated strain *Syn_Cn*SH^+^ [42]. Cultures were supplemented with different NiSO_4_ amounts to determine an optimal inducer concentration for *Cn*SH synthesis. *Cn*SH levels were verified via Western blots using a specific antibody for the HoxH subunit. *Cn*SH activities were analyzed via in-gel staining as well as via spectroscopic quantification of H_2_-driven NADH formation by cell extracts.

**Fig. 2:**
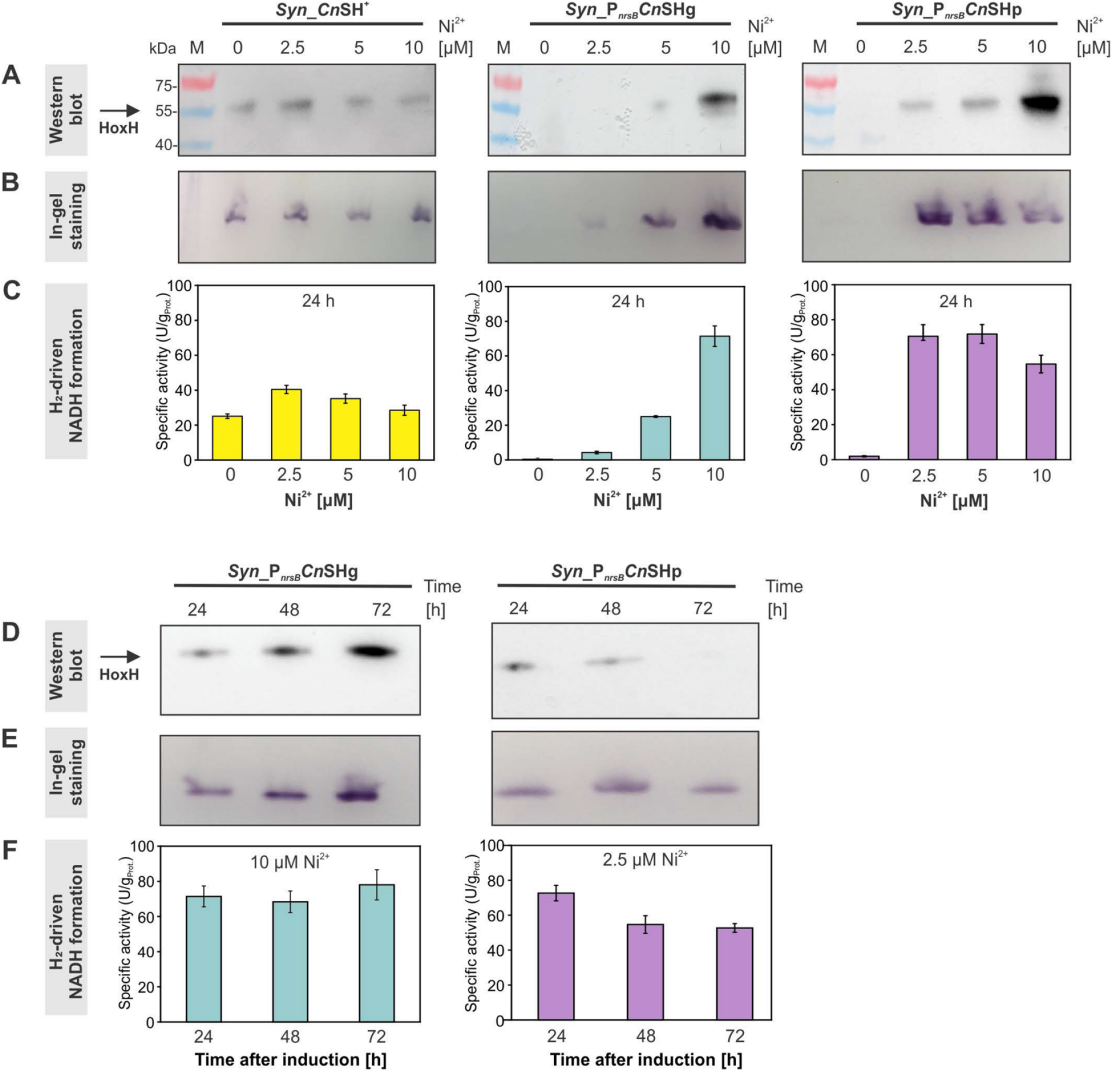
*Cn*SH abundance and specific activities in cell extracts of the recombinant *Synechocystis* strains *Syn_Cn*SH^+^, *Syn_P*_*nrsB*_*Cn*SHg, and *Syn_P*_*nrsB*_*Cn*SHp. All strains were grown photoautotrophically and treated with the given amounts of Ni^2+^, added as NiSO_4_, and harvested after the given time period. **A**–**C** Show the effect of different Ni^2+^ concentrations on *CnSH* gene expression and activity determined 24 h after Ni^2+^ addition, whereas panels **D**–**F** show respective time-dependent analyses after induction with Ni^2+^ concentrations found optimal in the experiments shown in panels **A**–**C**. **A**, **D** HoxH (55 kDa) was detected via Western Blot analysis of soluble protein separated by SDS-PAGE. Standard SDS-PAGE with Coomassie-blue staining was conducted as loading control (Fig. S3 and SF4). **B**, **E** In-gel activity staining was performed to detect H_2_-oxidizing *Cn*SH activity after native PAGE with soluble fractions. The staining relied on the coupling of H_2_-based NADH formation to NADH-mediated NBT reduction resulting in dark-colored bands. **C**, **F** Specific *Cn*SH activites in soluble extracts were quantified via NADH absorption in H_2_ saturated buffer. Data represent means ± standard deviations (n = 3)

In each case, Western blot signals were detected 24 h after NiSO_4_ addition, matching the molecular weight of HoxH (~ 55 kDa) (Fig. 2A). As expected, HoxH abundance in *Syn_Cn*SH^+^ was not influenced by the applied Ni^2+^ concentration. In contrast, HoxH could not be detected in *Syn*_*P*_*nrsB*_*Cn*SHg and *Syn*_*P*_*nrsB*_*Cn*SHp without Ni^2+^ addition, whereas increasing Ni^2+^ concentrations led to increasing HoxH levels (Fig. 2A).

In-gel activity staining confirmed that *Cn*SH activity in *Syn_Cn*SH^+^ did not depend on the applied Ni^2+^ concentration, whereas a dose-dependent response was observed for *Syn_P*_*nrsB*_*Cn*SHg (Fig. 2B), correlating with HoxH abundance (Fig. 2A). Interestingly, for *Syn_P*_*nrsB*_*Cn*SHp, activity-stained band intensities showed an inverse correlation with HoxH abundance (Fig. 2B). This was confirmed by activity assays with cell extracts, in which the highest *Cn*SH activity was found after induction with 2.5 and 5 μM Ni^2+^, whereas induction with 10 μM Ni^2+^ lead to a clearly reduced activity (Fig. 2C). Whereas *Syn_Cn*SH^+^ extracts showed a maximal activity of 40 U g_Prot_^−1^, extracts of optimally induced *Syn_P*_*nrsB*_*Cn*SHg (10 µM Ni^2+^) and *Syn_P*_*nrsB*_*Cn*SHp (2.5 µM Ni^2+^) reached ~ 70 U g_Prot_^−1^, a ~ 1.8-fold higher activity than *Syn_Cn*SH^+^.

*Cn*SH abundance and activity also were evaluated in a time-dependent manner for cells harvested 24, 48, and 72 h after induction with optimal Ni^2+^ concentrations (Fig. 2D–F). For *Syn_P*_*nrsB*_*Cn*SHg, HoxH signals were stably detected over this time period. Western blots and activity-stained gels even indicated an increase in HoxH levels with induction time, whereas *in-vitro*-activities remained constant at 70–80 U g_Prot_^−1^. *Syn_P*_*nrsB*_*Cn*SHp exhibited a similar maximal but less stable specific activity over time**.** HoxH thereby was found to be instable as visualized via Western blot analysis, in which the HoxH band almost completely vanished (Fig. 2D). No hydrogenase and respective activity were detected for the negative control *Syn*_∆*hox* (Fig. S5). In summary, a considerable *Cn*SH activity increase (1.8-twofold) was achieved in *Syn_P*_*nrsB*_*Cn*SHg and *Syn_P*_*nrsB*_*Cn*SHp. The lacking correlation between protein abundance and functionality of the hydrogenase complex, together with low stability over time indicates a non-optimal subunit ratio, rate of translation, absolute amount of *Cn*SH, or a possible *Cn*SH maturation issue in *Syn_P*_*nrsB*_*Cn*SHp. Therefore, *Syn_P*_*nrsB*_*Cn*SHg was selected for the following studies.

### Implementation of the maturation system of *C. necator* further improves specific *Cn*SH activity in *Synechocystis*

**Fig. 3:**
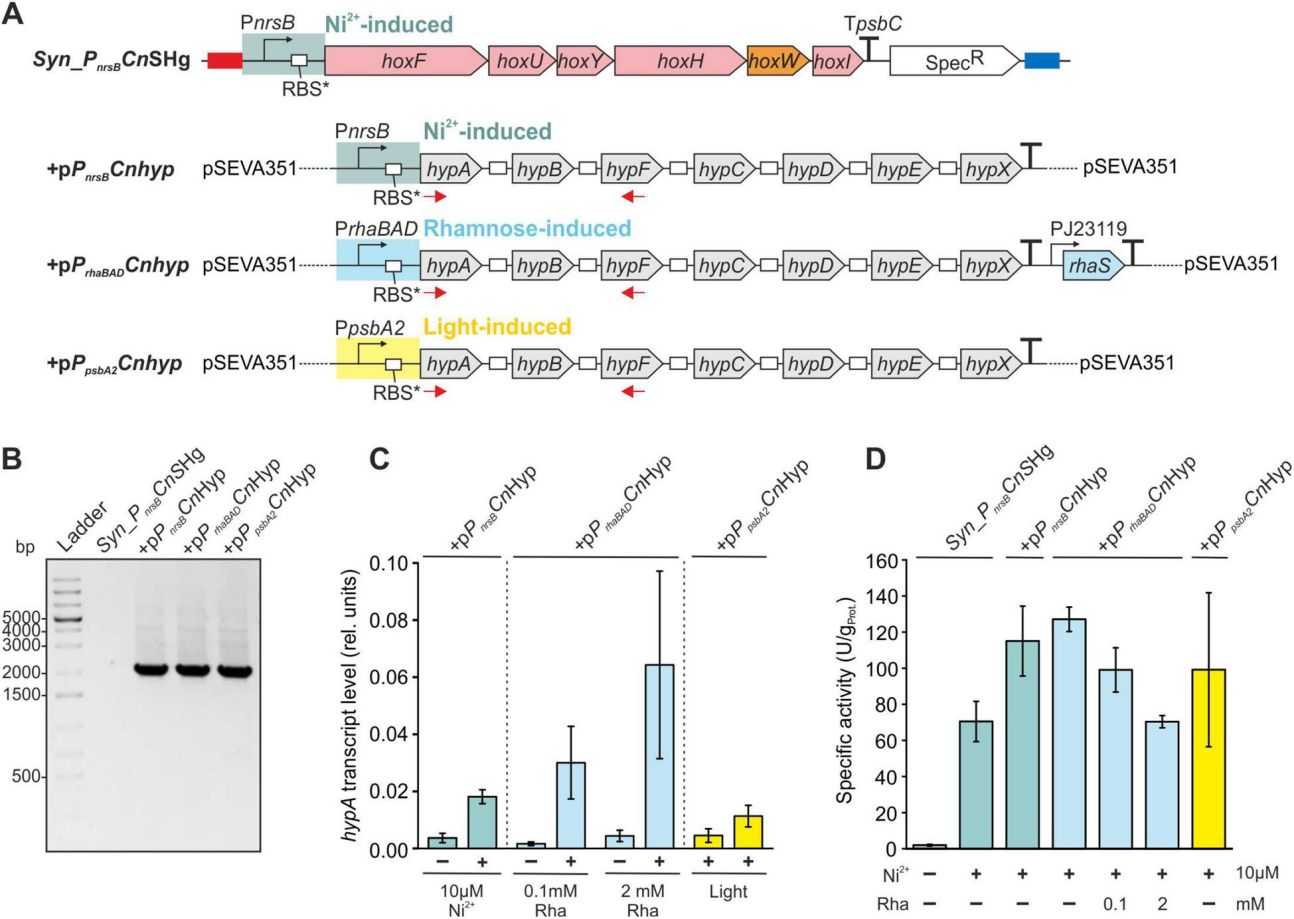
Co-expression of *hyp* genes from *C. necator* in strain *Syn_P*_*nrsB*_*Cn*SHg. **A** Schematic overview of the *hyp* gene constructs implemented in *Syn_P*_*nrsB*_*Cn*SHg. The synthetic *hyp* operon was combined with different promoters resulting in three different constructs based on the replicative pSEVA351 plasmid. The strain *Syn_P*_*nrsB*_*Cn*SHg is referred to with a + when containing a plasmid, which then is named after the + sign, to simplify nomenclature in this figure as well as in the text. **B** Verification of plasmid presence by colony PCR. *Syn_P*_*nrsB*_*Cn*SHg without plasmid was used as negative control. Clones were analyzed with primers targeting the region between *hypA* and *hypF* (primers represented by red arrows in panel B). Primer sequences are reported in Table S2. **C** Relative *hypA* transcript levels analyzed by qRT-PCR. Transcript abundance was determined before (−) and 24 h after (+) induction with Ni^2+^ for strain + p*P*_*nrsB*_*Cn*Hyp and rhamnose for strain + p*P*_*rhaBAD*_*Cn*Hyp. The same cultivation time points were used for the sampling of strain + p*P*_*psbA1*_*Cn*Hyp. Expression levels were normalized to the expression of the housekeeping gene *rnpB*. Means and standard deviations of biological duplicates (two independent clones) are shown, each measured in technical triplicates. **D** Specific *Cn*SH activities in soluble protein extracts prepared from cells grown for 48 h in presence of the given inducer concentrations. Given are mean values and standard deviations (n = 3)

**Fig. 4:**
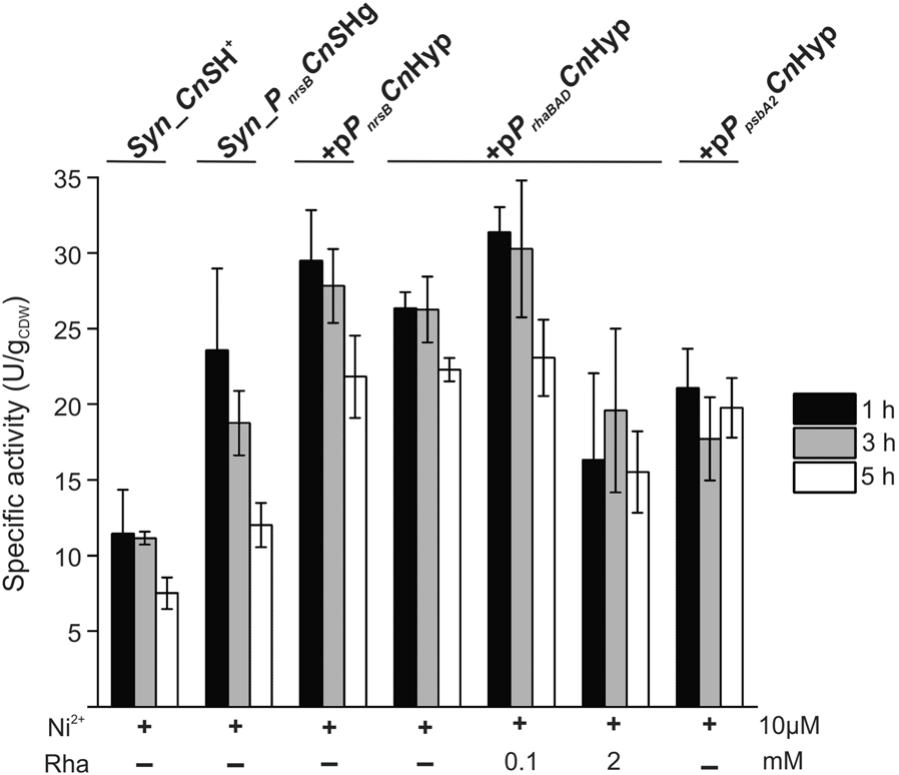
In vivo H_2_ consumption in photosynthetically active cells. Five mL cell culture of each strain were transferred into gas-tight 10 mL vials, flushed with a gas mixture composed of 20% H_2_, 10% CO_2_, and 70% N_2_, and incubated under illumination. The gas phase was analyzed via GC every at given time points to monitor H_2_ and O_2_ concentrations (Table S5). Specific activities in U g_CDW_^−1^ were calculated from H_2_ consumption as described in the methods section. Mean values and standard deviations (n = 3) are given

In *C. necator*, the minimal set of seven auxiliary maturases, encoded by the *hypA1B1F1CDEFX* genes, is responsible for the stepwise enzyme assembly and the incorporation of the Fe(CN)_2_CO cluster as well as the nickel ion into the HoxH apoprotein [8]. As next step, we assessed the impact of co-expressing auxiliary genes, i.e., those encoding the native hydrogenase maturation system (the Hyp proteins) from *C. necator* in strain *Syn_P*_*nrsB*_*Cn*SHg. For this purpose, we made use of a previously obtained polycistronic and codon-optimized *hypABFCDEFX* construct, in which the synthetic ribosomal binding site RBS* was placed upstream of every *hyp* gene for efficient translation initiation in *Synechocystis* [47]. We tested three different promoters: l-rhamnose-inducible *P*_*rhaBAD*_ from *E. coli* [3] and, from *Synechocystis*, Ni^2+^-dependent *P*_*nrsB*_ and light-regulated *P*_*psbA2*_ [13]. Utilization of *P*_*nrsB*_ for both *hox* and *hyp* genes aimed at similar expression strengths and induction times but prevents separate regulation of the two operons. In contrast, *P*_*rhaBAD*_ allows separate and tunable expression and P_*psbA2*_ a constitutive type of expression during photoautotrophic cultivation. Each *hyp* operon was combined with a transcriptional terminator. For the rhamnose inducible system, the *rhaS* cassette encoding the rhamnose-dependent regulator was placed downstream of the terminator [3]. *Syn_P*_*nrsB*_*Cn*SHg transformed with the resulting pSEVA351-based plasmids [43] led to the strains + p*P*_*nrsB*_*Cn*Hyp, + p*P*_*rhaBAD*_*Cn*Hyp, and + p*P*_*psbA2*_*Cn*Hyp (Fig. 3A). To simplify nomenclature in Figs. 3 and 4 as well as in the following text, the strain *Syn_P*_*nrsB*_*Cn*SHg is referred to with a + when containing a plasmid, which then is named after the + sign. Successful transformants were verified by colony PCR (Fig. 3B).

The designed strains were grown photoautotrophically and treated with the respective inducers for *hox* and *hyp* gene expression (see “Materials and methods” for details). Transcription of the *hyp* operon was analyzed by qRT-PCR targeting *hypA* prior to and 24 h after induction. As expected, Ni^2+^ or rhamnose addition significantly enhanced *hypA* transcript levels in strains + p*P*_*nrsB*_*Cn*Hyp or + p*P*_*rhaBAD*_*Cn*Hyp, resulting in 6- or 14–18-fold increases in *hypA* transcript levels, respectively (Fig. 3C). Plasmid p*P*_*psbA2*_*Cn*Hyp effected lower *hypA* transcript levels (Fig. 3C). It is important to note that the reversely transcribed complementary DNA (cDNA) was detected for every strain containing the *Cn_hyp* operon, even without induction (Fig. S6).

In *Synechocystis* strains expressing both the *hox* and *hyp* operons of *C. necator*, 13 genes overall, the synthesis/degradation of encoded proteins may present a high metabolic burden for the cells and consequently influence their growth behavior [70]. Recombinant gene expression, however, had only minor effects on phototrophic growth of *Synechocystis* (Fig. S7). Plasmid-free strains generally grew slightly faster than plasmid-containing strains in the absence as well as the presence of Ni^2+^ (Fig. S7A). Whereas p*P*_*psbA2*_*Cn*Hyp and p*P*_*nrsB*_*Cn*Hyp only had a minor effect on growth, simultaneous induction of the *hox* (Ni^2+^) and *hyp* (2 mM rhamnose) operons in strain + p*P*_*rhaBAD*_*Cn*Hyp had the most significant effect on growth, with a 20% reduced cell density 72 and 48 h after induction with Ni^2+^ and rhamnose, respectively (Fig. S7B).

The effect of recombinant *hyp* gene co-expression on *Cn*SH activity in *Synechocystis* was examined in vitro via monitoring of H_2_-driven NADH formation by soluble protein extracts (Fig. 3D). To this end, cells containing p*P*_*nrsB*_*Cn*Hyp, p*P*_*rhaBAD*_*Cn*Hyp, p*P*_*psbA2*_*Cn*Hyp, or no plasmid were treated with respective inducers in various combinations. A positive effect on *Cn*SH specific activity was found for strains + p*P*_*nrsB*_*Cn*Hyp and + p*P*_*rhaBAD*_*Cn*Hyp. In the latter case, non-induced (leaky) *hyp* operon expression led to a doubling compared to plasmid-free *Syn_P*_*nrsB*_*Cn*SHg, whereas low-level-induction (0.1 mM rhamnose) resulted in a 60% increase in *Cn*SH activity. This positive effect was abolished applying 2 mM rhamnose (Fig. 3D). This observation, along with the negative impact on growth caused by 2 mM rhamnose induction, suggests a metabolic burden of high-level *hyp* gene expression on the host, likely due to the high level of recombinant protein synthesis. The production of 13 proteins from *C. necator* inevitably intensifies competition for cellular resources involved in gene expression, protein synthesis, and folding, as well as the energy required for the hydrogenase maturation process and finally a possible H_2_ formation. The strain + p*P*_*psbA2*_*Cn*Hyp showed a highly variable *Cn*SH activity, which may reflect growth phase-dependent gene expression. Whereas expression of the entire *C. necator* maturase operon obviously can promote functional *Cn*SH synthesis in *Synechocystis*, high level *hyp* operon co-expression did not improve *Cn*SH activities and slightly affected growth.

### The yield of functional *Cn*SH complexes positively correlates with H_2_ oxidation in vivo

To determine if the enhanced enzymatic activity observed in vitro upon co-expression of the *hox* and *hyp* genes from *C. necator* correlates with increased H_2_ consumption in vivo, the activity of *Cn*SH was investigated for photosynthetically active cells. The H_2_ conversion rate of *Cn*SH in vivo primarily depends on three factors: (1) the abundance of functional *Cn*SH complexes, (2) the availability of H_2_ substrate, and (3) the presence of electron sinks capable of regenerating nicotinamide adenine dinucleotide (NAD^+^).

To characterize *Cn*SH activity in vivo, cells were transferred into sealed vials, flushed with a defined gas mixture (20% H_2_, 10% CO_2_, 70% N_2_), and incubated under continuous illumination for 6 or 8 h. H_2_ consumption and O_2_ evolution were monitored every 2 h (Table S5). In good agreement with the in vitro data, optimally induced *Syn_P*_*nrsB*_*Cn*SHg exhibited 1.8–2 times higher activity in vivo than *Syn_Cn*SH^+^ in the investigated time ranges. Provision of the plasmid p*P*_*rhaBAD*_*Cn*Hyp, both with and without 0.1 mM rhamnose induction, as well as of plasmid p*P*_*nrsB*_*Cn*Hyp, further promoted H_2_ oxidation activity up to twofold, especially in the long term, compared to plasmid-free *Syn_P*_*nrsB*_*Cn*SHg (Fig. 4).

Strain + p*P*_*rhaBAD*_*Cn*Hyp induced by 0.1 mM rhamnose exhibited the maximum H_2_ conversion rate of up to 32 U g_CDW_^−1^. Contrariwise, strain + p*P*_*rhaBAD*_*Cn*Hyp with 2 mM rhamnose or strain + p*P*_*sbA2L*_*Cn*Hyp led to similar H_2_ oxidation activities as obtained with plasmid-free *Syn_P*_*nrsB*_*Cn*SHg. Together with the in vitro results, these findings indicate an increased abundance of active hydrogenase upon fine-tuning of *hox* and *hyp* gene expression. The decrease in hydrogenase activity observed after 3 h (Fig. 4 and Table S6) can be related to limited sink availability for the H_2_-derived NADH pool.

## Discussion

In our previous work, we replaced the native hydrogenase of *Synechocystis* with the soluble, O_2_-tolerant [NiFe] hydrogenase from *C. necator* (*Cn*SH) and showed *Cn*SH activity in vivo and in vitro. Thereby, *Cn*SH was shown to be active in the presence of O_2_ and during photosynthetic water oxidation. The specific enzyme activity determined in cell-free extracts of *Syn_Cn*SH^+^, however, was up to two orders of magnitude lower than rates achieved in the native and heterotrophic hosts (Table 1) [41, 52, 53].

**Table 1:** Specific H_2_-oxidation activities of *Cn*SH in soluble fractions of different host strains

Strain	Condition	U g_Prot_^−1^	References
*C. necator* H16	CFE^a^	800–8000	[53] [52]
*E. coli*	CFE	1200	[52]
*P. putida*	PC^b^	150	[41]
*Syn_Cn*SH^+^	CFE	18 40^c^	[42] This work
*Syn_P*_*nrsB*_*Cn*SHg	CFE	80	This work
+ p*P*_*rhaBAD*_*Cn*Hyp	CFE	125	This work

^a^CFE: cell-free extract

^b^Permeabilized cells

^c^Optimized experimental procedure based on freshly grown cells instead of frozen cells pellets

In this context, it is important to note that strong and stable recombinant gene expression in cyanobacteria remains a challenge [29]. Previous studies indicated that expression levels are typically limited by slow transcription and translation, which are fundamentally controlled by the promoter and RBS elements, respectively [14].

Transcriptional control of the initially introduced *C. necator hox* operon relied on the native light-regulated *psb*A2 promoter and resulted in low expression levels. Recombinant gene expression using native *psb*A2 has been reported to suffer from strong dependence on light availability and growth status [19, 68]. We thus aimed for a well-controllable promoter with a wide dynamic range of induction. Among the native metal-inducible promoters, Ni^2+^-dependent *P*_*nrsB*_ has recently been characterized in *Synechocystis* as titratable and tight [13]. Together with *P*_*nrsB*_, the strong synthetic RBS* and a native terminator (T*psbC*) were used for *hoxFUYHWI* expression [24, 39, 64]. To improve cloning efficiencies and enable fast screening of genetic elements, we established a modular GoldenGate-type cloning system similar to the recently reported CyanoGate [62].

Both plasmid- and genome-based expression of the designed operon resulted in enhanced *Cn*SH synthesis and a doubling of the specific H_2_-oxidation activity (from 40 to 80 U g_Prot_^−1^). Gene expression with RSF1010-based plasmids [60] is known to be superior to genome-based expression, enabling higher gene copy numbers per cell (~ 30 plasmids vs 2–20 chromosome copies in *Synechocystis*) [65] and increased transcription during stationary phases [28], but suffers from a lower stability [30]. Indeed, higher *Cn*SH levels were achieved by plasmid-based expression, but maximally reached *Cn*SH activities were similar and differed regarding the optimal Ni^2+^ concentration necessary to achieve them. Further, only genome-based expression allowed stable hydrogenase production over 3 days post-induction, qualifying genome-based *Cn*SH expression in *Synechocystis* as superior.

The non-correspondence of *Cn*SH expression levels and activities (Fig. 3) indicates the presence of non-functional hydrogenases, which may be due to the absence of *C. necator* Hyp proteins. Indeed, it is well known that recombinant production of [NiFe] hydrogenase in heterologous hosts is challenging due to the complex maturation process [56]. Heterologous hydrogenase expression studies supported the hypothesis that the probability of obtaining a functional enzyme correlates with the abundance of homologous and heterologous Hyp proteins sharing a high degree of similarity [16]. Although *Synechocystis* and *C. necator* maturases show only 50–67% amino acid sequence homology [42], *Cn*SH maturation obviously is realized by the maturases of *Synechocystis*, but may suffer from a low efficiency. In studies conducted with *E. coli* as host strain, with an amino acid sequence identity between *C. necator* and *E. coli hyp* maturases of 18–45%, the omission of *C. necator* maturases severely reduced the recombinant hydrogenase activity [15, 52].

We firstly hypothesized that the absence of a *hypX* gene homolog in *Synechocystis* could have compromised aerobic *Cn*SH maturation. However, the introduction of *hyp*X only into *Syn*_*PnrsBCn*SHg did not influence the achieved hydrogenase activity, ruling out CO biogenesis as main limiting factor (Fig. S8). Therefore, it can be assumed that CO allocation is sufficient in *Synechocystis*. The introduction of the complete maturation system of *C. necator* into *Synechocystis* improved *Cn*SH expression level and activity. It is, however, important to note that a fine balance between hydrogenase and maturase gene expression seems essential to maximize functional *Cn*SH production. Elevated Hyp protein production or maturase activities appeared to negatively affect cell growth and hydrogenase activity so that their expression had to be quantitatively controlled. Thus, new genetic tools are needed to enhance and control heterologous gene expression. With the fine balance of recombinant multigene expression, the presence of *Cn*SH-dedicated auxiliary proteins enhanced its maturation efficiency in *Synechocystis* and, consequently, *Cn*SH activity. Further investigations, e.g., the separate expression of functional *Cn*Hyp complexes (HypCD, HypEF, HypAB) in *Synechocystis* and knockouts of endogenous maturases could be useful to determine the most efficient combination of maturases and to optimize heterologous *Cn*SH production in *Synechocystis*. As plasmid-based expression appeared to enable higher *Cn*SH levels in *Synechocystis*, genomic integration of *hyp* genes or the use of compatible plasmids [48] for *hox* and *hyp* gene expression may also be promising.

In the present study, we increased *Cn*SH activity in *Synechocystis* 3.1-fold (from 40 to 125 U g_Prot_^−1^) in a two-step approach—firstly, by improving protein synthesis via expression system engineering and secondly by the introduction of the *C. necator hyp* operon. The optimal expression conditions for the *C. necator* *hox* and *hyp* operons resulted in 60% higher H_2_ oxidation activity and enhanced in vivo stability compared to the expression of *hox* genes alone. This finding supports the hypothesis that the co-expression of *C. necator* maturases plays a crucial role in the formation of a functional recombinant hydrogenase complex. The rates obtained (125 U g_Prot_^−1^) are comparable to those achieved in heterotrophic hosts such as *P. putida* (~ 160 U g_Prot_^−1^) [41] (Table 1), paving the way for diverse applications of the O_2_-tolerant hydrogenase of *C. necator* in *Synechocystis*, e.g., photo-H_2_ production or H_2_ utilization to boost growth and/or biotransformation reactions.

## Supplementary Information

Supplementary Material 1: Fig. S1. Overview of the cloning strategy. Fig. S2. PCR to confirm the correct generation of *Syn_P*_*nrsB*_*Re*SHp strain. Fig. S3. SDS-PAGE loading control for Western blot analysis of expression levels with different inducer concentrations. Fig. S4. SDS-PAGE as loading control for Western Blot analysis of expression levels at different time points after induction. Fig. S5. Soluble cell-free extract from *Syn*_∆*hox* used as negative control. Fig. S6. (RT)-PCR targeting *hypX*. Fig. S7. Growth curves of not induced and induced strains expressing *C. necator* hydrogenase and maturases. Fig. S8. Characterization of *Syn_P*_*nsrB*_*Cn*SHg containing p*P*_*nrsB*_*Cn*HypX or p*P*_*rhaBAD*_*Cn*HypX. Table S1. Protocol for plasmid generation and propagation in *E. coli* DH5α. Table S2. Sequences of primers used in this study and of basal genetic elements implemented in our MoClo system. Table S3. List of plasmids generated in this work. Table S4. Strains used in this study. Table S5. Gas concentrations during in vivo H_2_ consumption assay. Table S6. H_2_ uptake rate during H_2_ consumption assay.

## Data Availability

No datasets were generated or analysed during the current study.
