# Supplementary material for: Co-expression of auxiliary genes enhances the activity of a heterologous O2-tolerant hydrogenase in the cyanobacterium Synechocystis sp. PCC 6803
Source: Biotechnol Biofuels Bioprod. 2025 Mar 28;18:41. doi: 10.1186/s13068-025-02634-5 (PMC11954184; doi:10.1186/s13068-025-02634-5)
Supplement: Supplementary file 1 — Supplementary Material 1: Fig. S1. Overview of the cloning strategy. Fig. S2. PCR to confirm the correct generation of Syn_PnrsBReSHp strain. Fig. S3. SDS-PAGE loading control for Western blot analysis of expression levels with different inducer concentrations. Fig. S4. SDS-PAGE as loading control for Western Blot analysis of expression levels at different time points after induction. Fig. S5. Soluble cell-free extract from Syn_∆hox used as negative control. Fig. S6. (RT)-PCR targeting hypX. Fig. S7. Growth curves of not induced and induced strains expressing C. necator hydrogenase and maturases. Fig. S8. Characterization of Syn_PnsrBCnSHg containing pPnrsBCnHypX or pPrhaBADCnHypX. Table S1. Protocol for plasmid generation and propagation in E. coli DH5α. Table S2. Sequences of primers used in this study and of basal genetic elements implemented in our MoClo system. Table S3. List of plasmids generated in this work. Table S4. Strains used in this study. Table S5. Gas concentrations during in vivo H2 consumption assay. Table S6. H2 uptake rate during H2 consumption assay. [file 13068_2025_2634_MOESM1_ESM.docx]

*Supplementary information*

Co-expression of auxiliary genes enhances the activity of a heterologous O_2_-tolerant hydrogenase in the cyanobacterium *Synechocystis* sp. PCC 6803

Lupacchini Sara^1^, Stauder Ron^2^, Opel, F.^2^, Klähn, S.^2^, Schmid Andreas^2^, Bühler Bruno^1^, Toepel Jörg^1^

^1^ Department of Microbial Biotechnology, Helmholtz Center for Environmental Research Leipzig

^2^ Department Solar Materials Biotechnology; Helmholtz Center for Environmental Research Leipzig

*Corresponding author: joerg.toepel@ufz.de


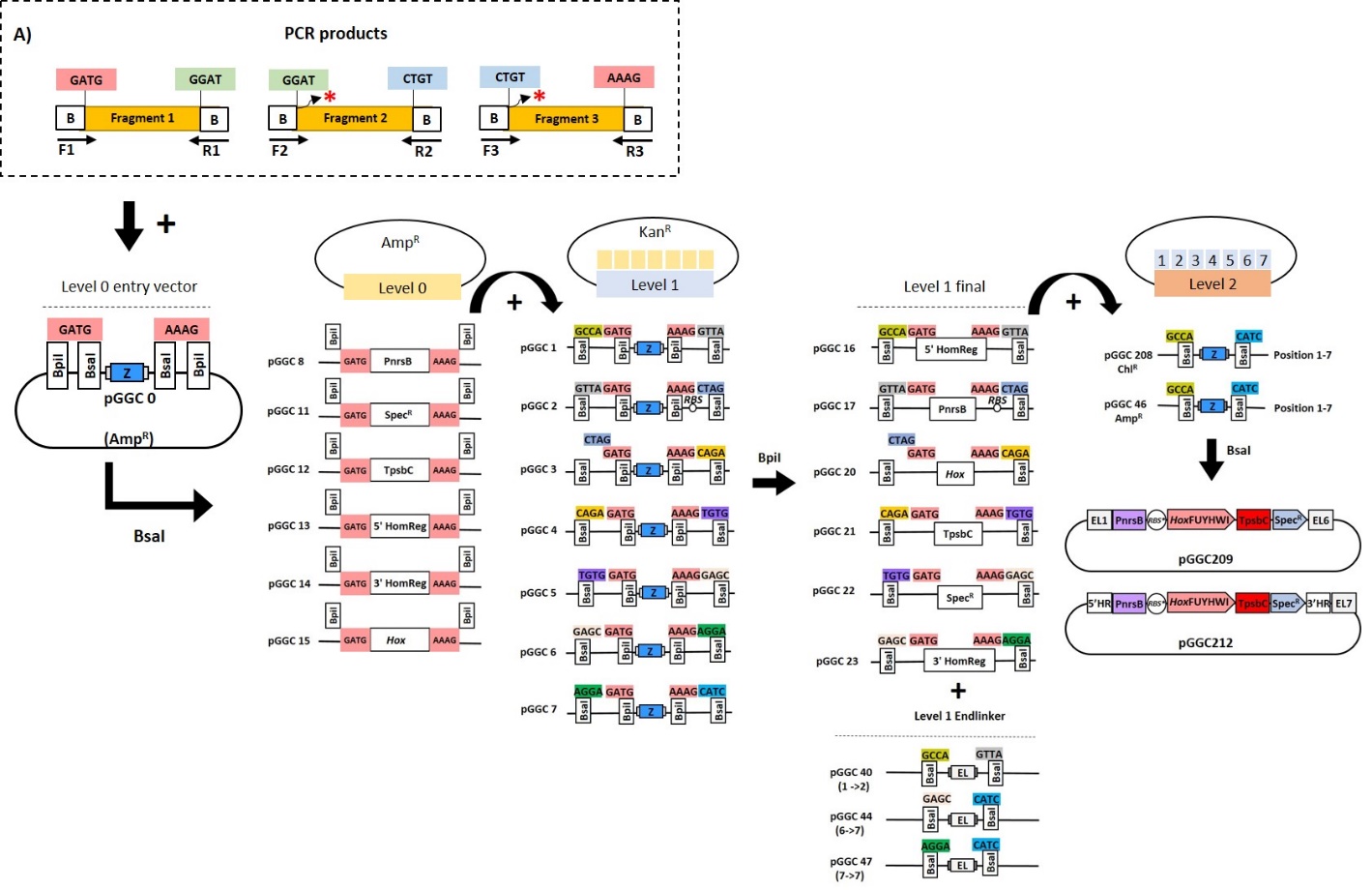


**Figure S1**. **Overview of the cloning strategy**. At first, each genetic element of interest was amplified either from *Syn*6803 genomic DNA or from existing vectors with primers flanked by a *Bsa*I recognition site containing 4 bp overhangs, in order to fit into level 0 vectors. If necessary, internal *Bpi*I/*Bsa*I restriction sites were removed using specific overlapping primers containing a single silent nucleotide exchange in the *Bsa*I/*Bpi*I recognition site (asterisk in **panel A**). Where necessary, the same procedure was applied to remove internal *Bpi*I/*Bsa*I restriction sites in the vector backbones, which are then defined as domesticated (Vasudevan *et al.*, 2019).

Level 0 generation: the domesticated pUC18 vector was amplified without *lacZ* cassette, using primers flanked by *Mlu*I and *Nco*I recognition sites and including the recognition sequences for *Bpi*I and *Bsa*I in inverse orientation relative to each other, producing the identical sticky end overhangs GATG and AAAG after cutting. The *lacZ* cassette was amplified individually with primers flanked also by *Mlu*I and *Nco*I restriction sites. Both purified fragments, the pUC18 backbone and the *lacZ* fragment, were digested with *Mlu*I and *Nco*I and ligated to generate the empty level 0 vector with *lacZ* cassette flanked up- and downstream with *Bpi*I*-Bsa*I restriction sites.

Level 1 generation: the pUK21 backbone was modified as described for level 0 vectors with the difference that the *lacZ* expression cassette was flanked up- and downstream with *Bsa*I-*Bpi*I restriction sites. *Bpi*I restriction results in the overhangs GATG and AAAG, which are matching with the overhangs carried by the genetic elements in level 0 vectors. The introduced *Bsa*I restriction sites generate new unique overhangs for each of the seven level 1 position plasmids generated. The level 1 position 2 vector was additionally modified by inserting the RBS sequence between the *Bpi*I and *Bsa*I restriction site upstream of the *lacZ* expression cassette. For level 1 end-linker generation, the pUK21 vector was amplified with primers flanked by *Bpi*I restriction sites. The forward primer also carried the end-linker sequence flanked with *Bsa*I restriction sites. The purified PCR product was digested with *Bpi*I and ligated to the final end-linker vectors. We generated various level 1 end-linker, to cover positions 1 to 7 in final level 2 vectors. Furthermore, we designed two types of level 2 vectors for the generation of replicative and genome integration vectors. As replicative vector, pSEVA 351 carrying an RSF1010 origin of replication (Martínez-García *et al.*, 2020) was modified as described for level 0 generation. In the vector backbone downstream of the T1 regulatory element, the T0 regulatory terminator element was replaced by the *lacZ* expression cassette flanked by two *Bsa*I restriction sites that produce the overhangs GCCA and CATC to assemble up to seven level 1 vectors. When less than seven genetic elements were combined in level 2 vectors, we used end-linkers to fill the remaining positions. As genome integration vector, the domesticated pBluescript II SK(+) backbone was modified by implementing a *lacZ* expression cassette as done for the replicative vector.


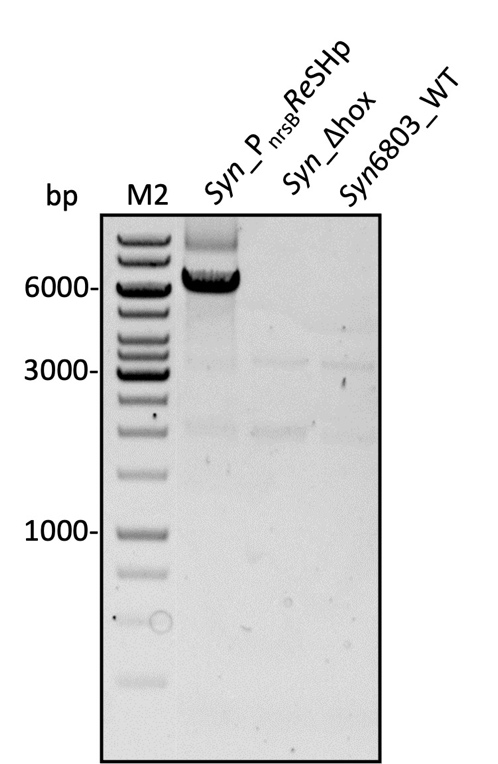


**Figure S2. PCR to confirm the correct generation of *Syn_P_nrsB_Cn*SHp strain.** The expected product size of ~6400 bp indicates the presence of the pGGC 209 plasmid carrying the *P_nrsB_Cn*SH *hox* operon (*Syn_P_nrsB_Cn*SHp). As negative control, the PCR also was performed using DNA from *Syn*_∆*hox* and *Synechocystis* WT. **M2**: GeneRuler 1 kb DNA Ladder, 250-10,000 bp (Thermo Fisher Scientific).


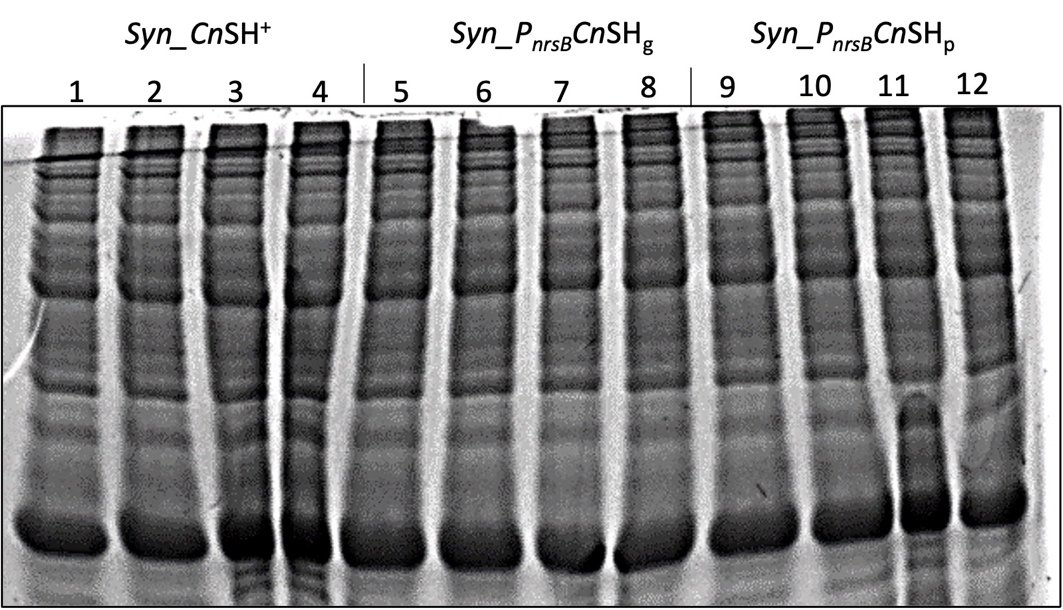


**Figure S3.** **SDS-PAGE loading control for Western blot analysis of expression levels with different inducer concentrations.** **1**: *Syn_Cn*SH^+^, 0 µM Ni^2+^; **2**: *Syn_Cn*SH^+^, 2.5 µM Ni^2+^; **3**: *Syn_Cn*SH^+^, 5 µM Ni^2+^; **4**: *Syn_Cn*SH^+^, 10 µM Ni^2+^; **5**: *Syn_P_nrsB_Cn*SHg, 0 µM Ni^2+^; **6**: *Syn_P_nrsB_Cn*SHg, 2.5 µM Ni^2+^; **7**: *Syn_P_n_*_rsB_*Cn*SHg, 5 µM Ni^2+^; **8**: *Syn_P_nrsB_Cn*SHg, 10 µM Ni^2+^; **9**: *Syn_P_nrsB_Cn*SHp, 0 µM Ni^2+^; **10**: *Syn_P_nrsB_Cn*SHp, 2.5 µM Ni^2+^; **11**: *Syn_P_nrsB_Cn*SHp, 5 µM Ni^2+^; **12**: *Syn_P_nrsB_Cn*SHp, 10 µM Ni^2+^.

*
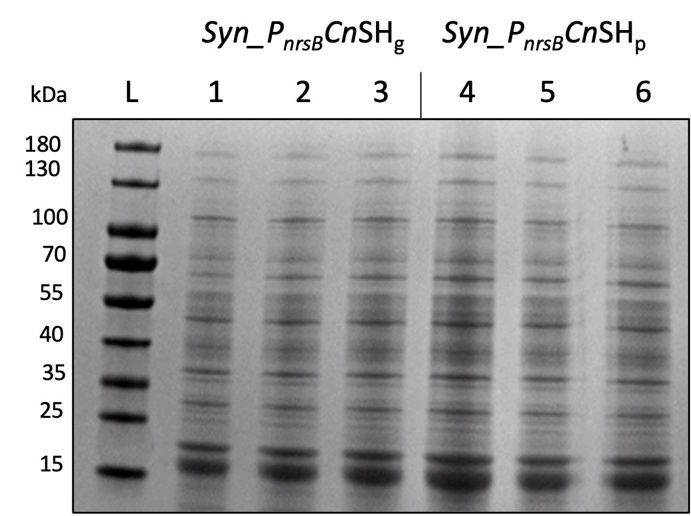
*

**Figure S4. SDS-PAGE as loading control for Western Blot analysis of expression levels at different time points after induction.** *Cn*SH abundance in *Syn_P_nrsB_Cn*SHg and *Syn_P_nrsB_Cn*SHp was analyzed in a time dependent manner after 24, 48, and 72 h after induction. **L**: Protein Ladder SM26616 (Thermo Fisher Scientific); **1**: *Syn_P_nrsB_Cn*SHg, 24 h after induction; **2**: *Syn_P_nrsB_Cn*SHg, 48 h after induction; **3**: *Syn_P_nrsB_Cn*SHg, 72 h after induction; **4**: *Syn_P_nrsB_Cn*SHp, 24 h after induction; **5**: *Syn_P_nrsB_Cn*SHp, 48 h after induction; **6**: *Syn_P_nrsB_Cn*SHp, 72 h after induction.


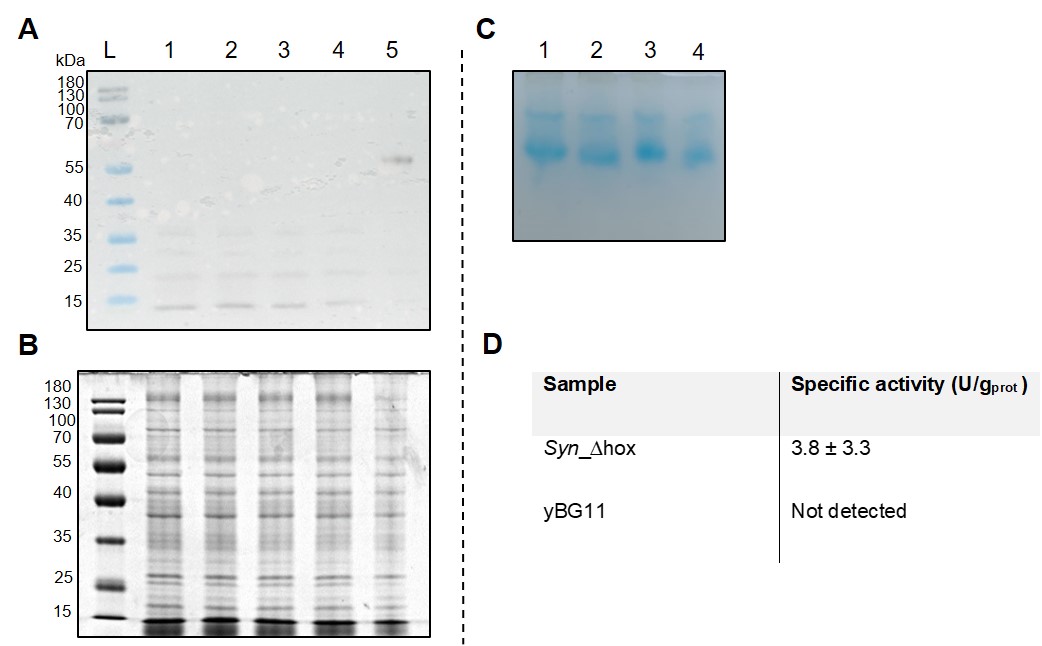


**Figure S5**. **Soluble cell-free extract from *Syn*_∆hox used as negative control.** (**A**) Western blot analysis for detection of Hox H and (**B**) denaturing SDS-PAGE analysis performed as loading control**. L:** Protein Ladder SM26616 (Thermo Fisher Scientific); **1**: Syn_∆*hox*, 0 µM Ni^2+^; **2**: Syn_∆*hox*, 2.5 µM Ni^2+^; **3**: Syn_∆*hox*, 5 µM Ni^2+^; **4**: Syn_∆*hox*, 10 µM Ni^2+^; **5**: *Syn_P_nrsB_CnSH*g, 10 µM Ni^2+^. (**C**) In gel activity staining: **1**: Syn_∆*hox*, 0 µM Ni^2+^; **2**: Syn_∆*hox*, 2.5 µM Ni^2+^; **3**: Syn_∆*hox*, 5 µM Ni^2+^; **4**: Syn_∆*hox*, 10 µM Ni^2+^. (**D**) *Syn*_∆hox and yBG11 medium used as negative control for H_2_-driven NAD^+^ reduction.


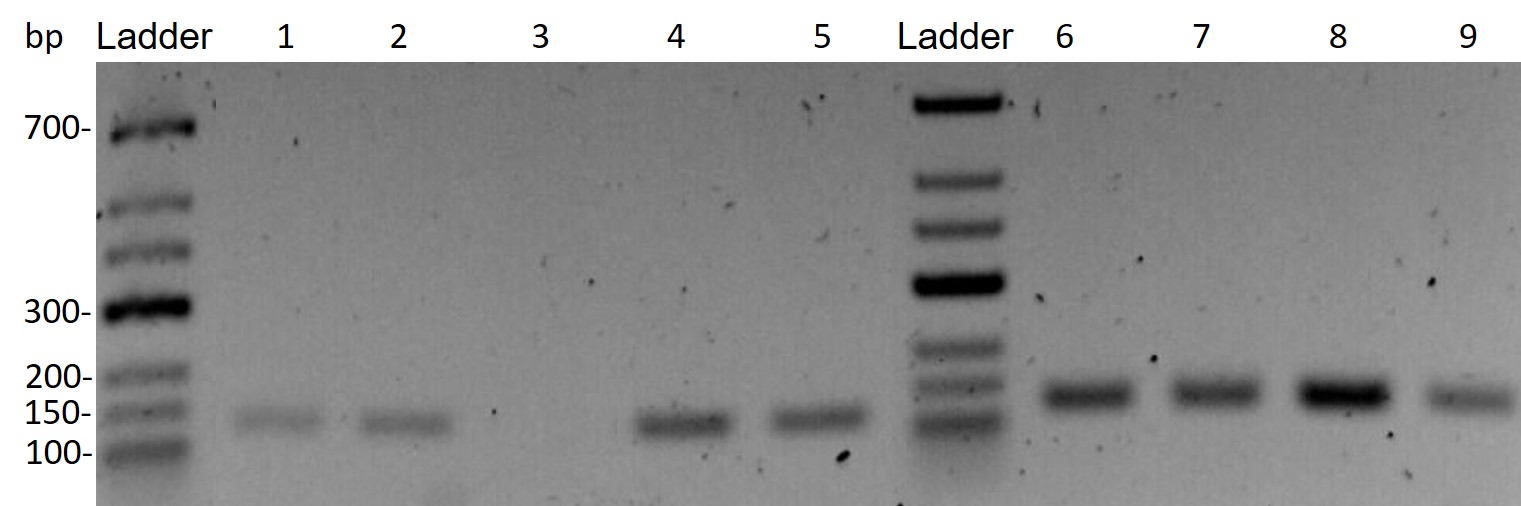


**Figure S6**: **(RT)-PCR targeting *hypX*.** The *hypX* mRNA was reverse transcribed for every strain analyzed, before and 24 h after induction, as illustrated by the band at ~124 bp. The negative control (line 3), which is not harboring the *Cn_hyp* plasmid, does not show *hypX* reverse transcription. Ladder: GeneRuler^TM^ Low Range; **1**: +p*P_nrsB_Cn*Hyp not induced; **2**: +p*P_nrsB_Cn*Hyp: 10µM Ni^2+^; **3**: *Syn_P_nrsB_Cn*SHg only (negative control); **4**: +p*P_rhaBAD_Cn*Hyp not induced; **5**: +p*P_rhaBAD_Cn*Hyp induced with 0.1mM rhamnose; **6**: +p*P_rhaBAD_Cn*Hyp not induced; **7**: +p*P_rhaBAD_Cn*Hyp induced with 2 mM rhamnose; **8**: p*P_psbA2_Cn*Hyp; **9**: p*P_psbA2_Cn*Hyp (24 h later).


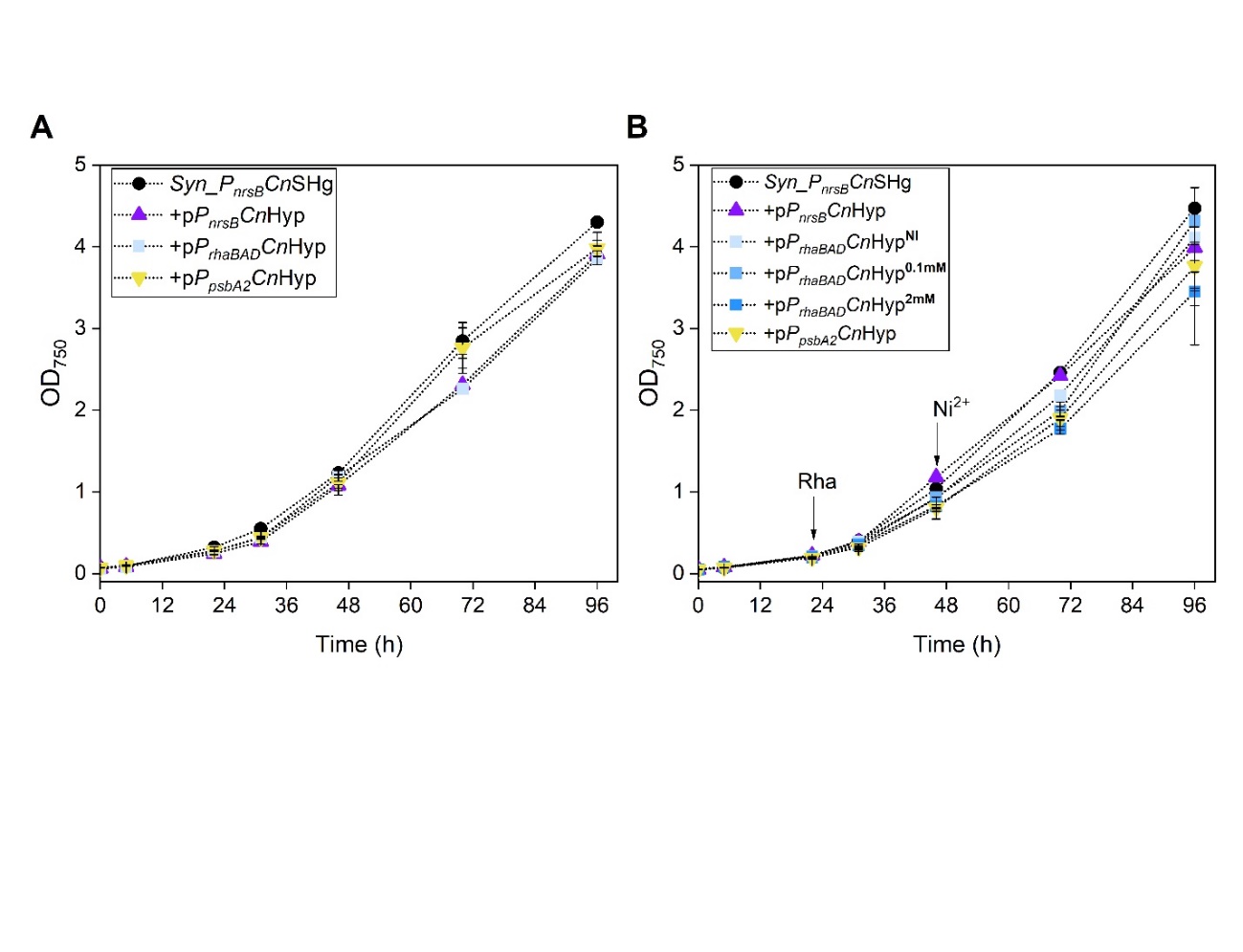


**Figure S7. Growth curves for not induced (A) and induced (B) strains expressing *C. necator* hydrogenase and maturases.** Cultures were grown under phototrophic conditions (50 µE, 2% CO_2_) and were supplemented with Ni^2+^ and rhamnose for *hox* and *hyp* operon expression as indicated. Panel (**A**) shows growth curves for *Syn_P_nrsB_Cn*SHg containing p*P_nrsB_Cn*Hyp, p*P_psbA2_Cn*Hyp, p*P_rhaBAD_Cn*Hyp or no plasmid without any nickel and rhamnose supplementation. Panel (**B**) compares growth of the same set of strains provided with 10 µM Ni^2+^ and, for strains carrying p*P_rhaBAD_Cn*Hyp, 0.1 mM, 2 mM, or no rhamnose (only nickel, Ni). Data represent means ± standard deviations (n = 2).


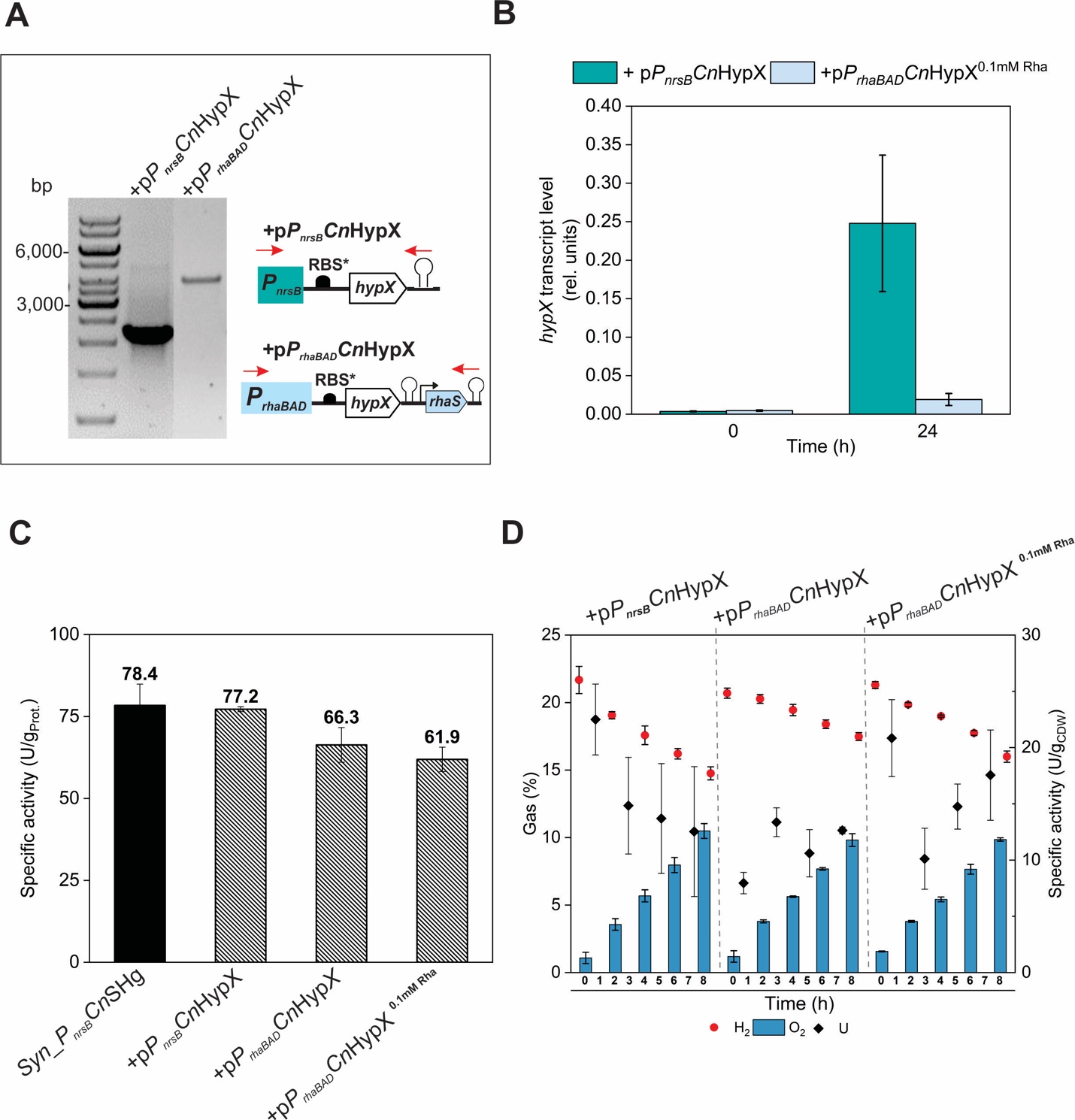


**Figure S8. Characterization of *Syn_P_nsrB_Cn*SHg containing p*P_nrsB_Cn*HypX or p*P_rhaBAD_Cn*HypX**. (**A**) Genetic setup of *hypX* operons. Red arrows indicate the binding sites of primers used to verify successful transformation of via colony-PCR as shown by the agarose gel. The expected product sizes of ~2100 and ~4200 bp indicate the presence of the pGGC 243 and 244 plasmids in *Syn_P_nrsB_Cn*SHg, respectively, giving +p*P_nrsB_Cn*HypX and +p*P_rhaBAD_Cn*HypX strains. (**B**) qRT-PCR of *hypX* determined before (0 h) and 24 h after induction with nickel for +p*P_nrsB_Cn*HypX and rhamnose for +p*P_rhaBAD_Cn*HypX strains. Shown are the averages and standard deviations of biological duplicates. (**C**) Specific hydrogenase activities determined *in vitro* using soluble protein extract of each strain. Ni^2+^ (10 µM) was supplemented 24 h before the assay to each strain. Shown are mean values and standard deviations (n=3). (**D**) Hydrogen consumption by whole cells of different HypX containing strains. Of each culture, 5 ml were transferred into 20 ml gas-tight vials and incubated for 8 h under illumination with 20% H_2_, 10% CO_2_, and 70% N_2_. Ni^2+^ (10 µM) was supplemented 24 h before the assay to each strain*. Syn_P_nrsB_Cn*SHg (positive control) is shown in **Fig. 3A**. Shown are mean values and standard deviations (n=3).

**Table S1. Protocol for plasmid generation and propagation in *E. coli* DH5α.** Golden Gate assembly reactions were performed with the restriction enzymes *BsaI*-HF®v2 (New England Biolabs) or *Bpi*I FD (Thermo Fisher Scientific) and T4 DNA Ligase HC (Promega). For each ligation reaction mixture, 2 µl were transformed into *E. coli* DH5α via electroporation (2500 V, 5 ms) followed by plating on LB-agar supplemented with IPTG (1 mM), Xgal (4 0 mg/mL), and the antibiotic necessary for selection. Verification of positive clones was done through blue-white screening and sequencing (Julin, 2018).

| **Component** | **Level 0** | **Level 1** | **Level 2** |
| --- | --- | --- | --- |
| Source of DNA inserts, restriction digested | PCR product (40 fmol) | Level 0 vector (40 fmol) | Level 1 vectors  (pos. 1 to 7) (40 fmol) |
| Vector DNA, restriction digested | Level 0 vector (40 fmol) | Level 1 vector (40 fmol) | Level 2 vector (80 fmol) |
| NEB T4 DNA ligase Buffer (10x) | 1x | 1x | 1x |
| Enzyme | *Bsa*I-HFv2 (10 U) | *Bpi*I FD (0.5 µl) | *Bsa*I-HFv2 (20 U) |
| T4 DNA Ligase | 6 U | 6 U | 6 U |
| Nuclease-free water | To fill up to a total volume of 20 µl | | |

| **Incubate the reactions at:** | |
| --- | --- |
| **Level 0/1** | **Level 2** |
| 37°C for 2 h  50°C for 5 min  80°C for 5 min | 37°C for 2 min  X50  16°C for 5 min  50°C for 5min  80°C for 5 min |
| 4°C hold | |

**Table S2.** **Sequences of primers used in this study and of basal genetic elements implemented in our MoClo system.** **A**. Primers designed to generate MoClo vectors **B.** Primers designed for cloning in Level 0. GGTCTC is the *Bsa*I recognition site. The designed 4 bp overhangs used to enter level 0 are given in yellow and primer binding regions in bold. **C.** Primers used for colony PCR and qRT-PCR.

| **A) Primer name** | **Sequence 5´ -> 3´** | **Primer target** |
| --- | --- | --- |
| P_LacZ_for | AAAACGCGTGCGCAACGCAATTAATGTGAG | *lacZ* fragment |
| P_LacZ_rev | AAACCATGGCTATGCGGCATCAGAGCAGA |  |
| P_BB_pUC18_for | AAACCATGGGGTCTCTAAAGAAGTCTTCTTAAGCCAGCCCCGACA | Level 0 backbone with flanking *Bpi*I and *Bsa*I sites |
| P_BB_pUC18_rev | AAAACGCGTGGTCTCTCATCTAGTCTTCTCACTGCCCGCTTTCCA |  |
| P_pUC18_BsaI_remove_for | AAAGGTCTCAGGCTCTCGCGGTATCATTG | Level 0 vector, removal of internal BsaI site |
| P_pUC18_BsaI_remove_rev | AAAGGTCTCAAGCCACGCTCACCGGCTCCAG |  |
| p_BB_pUK21_for Pos1 | AAACCATGGGAAGACAAAAAGGTTAAGAGACCGGCGGGTGTGGTGGTTA | Level 1 Position 1 backbone with flanking *Bsa*I and *Bpi*I sites |
| p_BB_pUK21_rev Pos1 | AAAACGCGTGAAGACTTCATCTGGCTGAGACCATTGCGTTGCGCTCACTG |  |
| p_BB_pUK21_for Pos2 | AAACCATGGGAAGACTTAAAGAGGAGAAATACTAGTGAGACCGGCGGGTGTGGTGGTTA | Level 1 Position 2 backbone with flanking *Bsa*I and *Bpi*I sites |
| p_BB_pUK21_rev Pos2 | AAAACGCGTGAAGACAACATCTAACTGAGACCATTGCGTTGCGCTCACTG |  |
| p_BB_pUK21_for Pos3 | AAACCATGGGAAGACAAAAAGCAGAAGAGACCGGCGGGTGTGGTGGTTA | Level 1 Position 3 backbone with flanking *Bsa*I and *Bpi*I sites |
| p_BB_pUK21_rev Pos3 | AAAACGCGTGAAGACAACATCTAGTGAGACCATTGCGTTGCGCTCACTG |  |
| p_BB_pUK21_for Pos4 | AAACCATGGGAAGACAAAAAGTGTGAGAGACCGGCGGGTGTGGTGGTTA | Level 1 Position 4 backbone with flanking *Bsa*I and *Bpi*I sites |
| p_BB_pUK21_rev Pos4 | AAAACGCGTGAAGACAACATCTCTGTGAGACCATTGCGTTGCGCTCACTG |  |
| p_BB_pUK21_for Pos5 | AAACCATGGGAAGACAAAAAGGAGCAGAGACCGGCGGGTGTGGTGGTTA | Level 1 Position 5 backbone with flanking *Bsa*I and *Bpi*I sites |
| p_BB_pUK21_rev Pos5 | AAAACGCGTGAAGACAACATCCACATGAGACCATTGCGTTGCGCTCACTG |  |
| p_BB_pUK21_for Pos6 | AAACCATGGGAAGACAAAAAGAGGAAGAGACCGGCGGGTGTGGTGGTTA | Level 1 Position 6 backbone with flanking *Bsa*I and *Bpi*I sites |
| p_BB_pUK21_rev Pos6 | AAAACGCGTGAAGACAACATCGCTCTGAGACCATTGCGTTGCGCTCACTG |  |
| EL 1-->2_for | AAAGAAGACAGAATTGGTCTCAGCCATCGGTCACATGTGCATCCTCGATCTCAGTTATGAGACCGTGTGGTGGTTACGCGCAG | Level 1 end-linkers with flanking *Bsa*I sites |
| EL 6-->7_for | AAAGAAGACAGAATTGGTCTCAGAGCTCGGTCACATGTGCATCCTCGATCTCACATCTGAGACCGTGTGGTGGTTACGCGCAG |  |
| EL 7-->7_for | AAAGAAGACAGAATTGGTCTCAACGTTCGGTCACATGTGCATCCTCGATCTCACATCTGAGACCGTGTGGTGGTTACGCGCAG |  |
| EL_rev | AAAGAAGACATAATTGCGTTGCGCTCACTG |  |
| P_pBl_BsaI_rem_for | AAAGGTCTCAGTGGCTCTCGCGGTATCA | Level 2 integrative vector, removal of internal *Bsa*I site |
| P_pBl_BsaI_rem_rev | AAAGGTCTCACCACGCTCACCGGCTCCAGA |  |
| P_BB_pBl_for Pos 7 | AAACCATGGGGTCTCTCATCGACGCGCCCTGTAGCG | Level 2 integrative backbone with flanking *Bsa*I sites |
| P_BB_pBl_rev Pos 7 | AAAACGCGTGGTCTCATGGCTCACTGCCCGCTTTCCAG |  |
| p_BB_pSEVA351_for | AAACTCGAGGGTCTCTCATCGGGTCCCCAATAATTACG | Level 2 replicative backbone with flanking *Bsa*I sites |
| p_BB_pSEVA351_rev | AAAACGCGTGGTCTCATGGCGGCATCAAATAAAACGAAAGG |  |
| **B) Primer name** | **Sequence 5´ -> 3´** | **Primer target** |
| P_Hox-Gen_F1 | AAAGGTCTCGGATG**ATGGATAGTCGTATCACGACAATACT** | *C. necator hox*-operon out of plasmid pGE3382 |
| P_Hox-Gen_R1 | AAAGGTCTCGGTTT**CAATCACTTGTTCGGGC** |  |
| P_Hox-Gen_F2 | AAAGGTCTCGAAAC**CATCGTCGACTCCAG** |  |
| P_Hox-Gen_R2 | AAAGGTCTCGGTTT**CAACGTTGTTGACGC** |  |
| P_Hox-Gen_F3 | AAAGGTCTCGAAAC**CTTTGCCGCCGT** |  |
| P_Hox-Gen_R3 | AAAGGTCTCTCGAT**ACCCGCACCGTAC** |  |
| P_Hox-Gen_ F4 | AAAGGTCTCTATCG**AAGGGCCTGAATGTCG** |  |
| P_Hox-Gen_R4 | AAAGGTCTCCGATA**CCATCATGTCCACCTCG** |  |
| P_Hox-Gen_F5 | AAAGGTCTCGTATC**GCGCTTTCCGTACC** |  |
| P_Hox-Gen_R5 | AAAGGTCTCGTCCT**CTCCTTCCAGCGC** |  |
| P_Hox-Gen_F6 | AAAGGTCTCGAGGA**CAAATGAGAGCCC** |  |
| P_Hox-Gen_R6 | AAAGGTCTCAACAC**GTTGCGCATCG** |  |
| P_Hox-Gen_F7 | AAAGGTCTCCGTGT**TCGAGCTGAAAGATTGTC** |  |
| P_Hox-Gen_R7 | AAAGGTCTCCGATA**CCACCAGCGGCAT** |  |
| P_Hox-Gen_F8 | AAAGGTCTCGTATC**GGTCTTTGACGCGG** |  |
| P_Hox-Gen_R8 | AAAGGTCTCGCTTT**CTAACCCCGTCCCCTCC** |  |
| P_PnrsB_F | AAAGGTCTCGGATG**TTCCACCAGCAAAATTCGCA** | *P_nrsB_* promotor out of gDNA from *Synechocystis sp.* PCC6803 |
| P_PnrsB_R | AAAGGTCTCGCTTT**AATTGGGAATTTGTCCAAGATTTT** |  |
| P_TpsbC_F | AAAGGTCTCGGATG**ATTGAGACTTTTCTGATTTTGCAAAGG** | T*_psbC_* terminator out of gDNA from *Synechocystis sp.* PCC6803 |
| P_TpsbC_R | AAAGGTCTCGCTTT**AACACCAGCGGGGAAAGG** |  |
| P_spec_F1 | AAAGGTCTCGGATG**TCGCGCAGGCTGGG** | Spectinomycin resistance cassette out of plasmid pHP45Ω |
| P_spec_R1 | AAAGGTCTCCGGAA**GTCCTCGGCCG** |  |
| P_spec_F2 | AAAGGTCTCCTTCCG**ATCTCCTGAAGCCAG** |  |
| P_spec_R2 | AAAGGTCTCGGTTT**CCACGCATCGTCAG** |  |
| P_spec_F3 | AAAGGTCTCGAAAC**CGAAACCTTGCGCTC** |  |
| P_spec_R3 | AAAGGTCTCGCTTT**CTAGATTTTAATGCGGATGTTGCGA** |  |
| P_3´HomReg_F1 | AAAGGTCTCGGATG**GCATCACCGAGGGCATATCTAG** | 3’ flanking region out of gDNA from *Synechocystis sp.* PCC6803 |
| P_3´HomReg_R1 | AAAGGTCTCGTTTC**TGCCTCAGTTTTGGCT** |  |
| P_3´HomReg_F2 | AAAGGTCTCAGAAA**CCATAGTTTAAAGGGCTAGTTG** |  |
| P_3´HomReg_R2 | AAAGGTCTCGCTTT**CACGGCACTGGCACTCT** |  |
| P_5' HomReg_F | AAAGGTCTCGGATG**GTTTTTATCTGCCAGTGAAGCCC** | 5’ flanking region out of gDNA from *Synechocystis sp.* PCC6803 |
| P_5' HomReg_R | AAAGGTCTCGCTTT**GATAAAAGATGATTGGGG** |  |

| P_HypA1_F | AAAGGTCTCGGATGCACGAATTGTCCTTGGCC | *C. necator* codon optimized *hyp*-operon out of pHySe_Hox_Hyp |
| --- | --- | --- |
| P_HypX_R | AAAGGTCTCGCTTTTTATTTTTCGAACTGCGGGTGGCT |  |
| P_PrhaS_F | AAAGGTCTCGGATGCCACAATTCAGCAAATT | *P_J23119_::rhaS out of P_rhaBAD_ pSHDY_P_rhaBAD_::mVenus _PJ23119-rhaS* |
| P_PrhaS_R | AAAGGTCTCGCTTTTTCATTACGACCAGTCTA |  |
| P_TrhaS_F | AAAGGTCTCGGATGTTGACAGCTAGCTCAGT | *P_J23119_::rhaS, out of P_rhaBAD_ pSHDY_P_rhaBAD_::mVenus _PJ23119-rhaS* |
| P_TrhaS_R | AAAGGTCTCGCTTTTATAAACGCAGAAAGGCCCA |  |
| P_pGH1_hypX_F1 | AAAGGTCTCGGATGCGCATATTGCTCCTCACC | *C. necator hypX* out of megaplasmid pGH1 |
| P_pGH1_hypX_R1 | AAAGGTCTCATCATCTTCCCAGTACCGGAACT |  |
| P_pGH1_hypX_F2 | AAAGGTCTCGATGATTCCGGCGC |  |
| P_pGH1_hypX_StrpTag_R2 | AAAGGTCTCGCTTTTTATTTTTCGAACTGCGGGTGGCTCCAAGCAGATCGTTTCCCCGC |  |
| P_PsbA2_F | AAAGGTCTCGGATGGTTCCAGTGGATATTTGCTGG | *P_psbA2_* out of gDNA from *Synechocystis sp.* PCC6803 |
| P_PsbA2_R | AAAGGTCTCGCTTTATGTATTTGTCGATGTTCAGATTGG |  |

| **C) Primer name** | **Sequence 5´ -> 3´** | **Primer target** |
| --- | --- | --- |
| PCC6803_F | CCTGGTTTAGGCTCTCCC | Targeting *Syn*6803 genome region upstream and downstream of *Cn*Hox operon integration site |
| CnHox__R | CCCCAGAGCAAATTGACTTGATTCA |  |
| Pseva_F | CTAGCGCAGCGAATAGAC | Targeting pSEVA backbone upstream the *Cn*Hox operon in *Syn*_*P_nrsB_Cn*SHp |
| Term_R | AACACCAGCGGGGAAAGG | Targeting T*_psbC_* terminator placed at the end of *Cn*Hox operon in *Syn*_*P_nrsB_Cn*SHp |

| P_HypA1_F | AAAGGTCTCGGATGCACGAATTGTCCTTGGCC | *hypA1* gene in the *hyp* operon |
| --- | --- | --- |
| PS37_R | ATTGGCGTTCCAACATGG | *hypF1* gene in the *hyp* operon |
| #43_F | AAAGGTCTCGGATGTTCCACCAGCAAAATTCGCA | *P_nrsB_* in pGGC 243 |
| # 38_R | AAAGGTCTCGCTTTAACACCAGCGGGGAAAGG | *T_psbC_* in pGGC 243 |
| # 62_F | AAAGGTCTCGGATGCCACAATTCAGCAAATT | *P_rhaBAD_* in pGGC 244 |
| # 70_R | AAAGGTCTCGCTTTTATAAACGCAGAAAGGCCCA | *rhaS* cassette in pGGC 244 |
| # 295_F | GCCTGGTGTTTGCAATGTAACG | *hypA1* gene in the *hyp* operon |
| # 296_R | CGGGACACCAGTGATCTTCC |  |
| # 297_F | GCCACAGAAAAATACCGCCC | *rnpB* houskeeping gene in *Syn*6803 genome |
| # 298_R | CACCTTTGCACCCTTACCCT |  |
| # 308_F | CATTCCGTGACCGAGGAGG | *hypX* gene in the *hyp* operon |
| # 309_R | GATCGCCGACAATGCCAG |  |
| # 304_F | GGCCCAGTATCGAGAGGAAG | *hypX* gene in the *hypX* operon from pGH1 |
| # 305_R | GTGCCTGCAACTCTTGTGCC |  |

**Table S3.** **List of plasmids used and generated in this work.**

|  |  |  | |  | |  | |  | |  |  |  | | |  | |  |  |
| --- | --- | --- | --- | --- | --- | --- | --- | --- | --- | --- | --- | --- | --- | --- | --- | --- | --- | --- |
|  | **Vector** | **Level** | | **5' overhang** | | **3' overhang** | | **Backbone** | | **Selection** | **Modified** | **Characteristics** | | | **Reference** | |  |  |
|  | **pGE3382** |  | |  | |  | |  | |  | no | Carrying *C. necator* *hox*-operon *hoxFUYHWI* and *C. necator hyp* genes *hypB2F2D1E1X* | | | (Lonsdale *et al.*, 2015) | |  |  |
|  | **pHP45Ω** |  | |  | |  | |  | |  | no | Spectinomycin resistance cassette | | | (Prentki and Krisch, 1984) | |  |  |
|  | pHySe_Hox_Hyp |  | |  | |  | |  | |  | no | pHySe_Hox backbone,  *P_nrsB_*::*hypA1B1F1CDEX*^codon, Strep^ | | | (Opel *et al.*, 2023) | |  |  |
|  | pSHDY_*P_rhaBAD_::mVenus _PJ23119-rhaS* |  | |  | |  | |  | |  | no | *P_J23119_::rhaS, P_rhaBAD_* | | | (Behle *et al.*, 2020), (Opel *et al.*, 2023) | |  |  |
|  | **pGGC 0** | 0 | | GATG | | AAAG | | pUC18 | | Amp^R^ | yes | Level 0 empty entry vector based on pUC18 vector with additional integrated *BpiI/BsaI* restriction sites flanking *lacZα* | | | This study | |  |  |
|  | **pGGC 1** | 1 | | GCCA | | GTTA | | pUK21 | | Kan^R^ | yes | Level 1 position 1 till 6 empty entry vectors based on pUC19 vector with additional integrated *BsaI/BpiI* restriction sites flanking *lacZα* | | | This study | |  |  |
|  | **pGGC 2** | 1 | | GTTA | | CTAG | | pUK21 | | Kan^R^ | yes |  |  |  | This study | |  |  |
|  | **pGGC 3** | 1 | | CTAG | | CAGA | | pUK21 | | Kan^R^ | yes |  |  |  | This study | |  |  |
|  | **pGGC 4** | 1 | | CAGA | | TGTG | | pUK21 | | Kan^R^ | yes |  |  |  | This study | |  |  |
|  | **pGGC 5** | 1 | | TGTG | | GAGC | | pUK21 | | Kan^R^ | yes |  |  |  | This study | |  |  |
|  | **pGGC 6** | 1 | | GAGC | | AGGA | | pUK21 | | Kan^R^ | yes |  |  |  | This study | |  |  |
|  | **pGGC 40** | 1 | | GCCA | | GTTA | | pUK21 | | Kan^R^ | yes | End-linker level 1 spanning position 1 till 2 based on pUC19 vector with additional integrated *BsaI/BpiI* restriction sites flanking end-linker sequence TCGGTCACATGTGCATCCTCGATCTCA | | | This study | |  |  |
|  | **pGGC 43** | 1 | | GAGC | | CATC | | pUK21 | | Kan^R^ | yes | End-linker level 1 spanning position 5 till 7 based on pUC19 vector with additional integrated *BsaI/BpiI* restriction sites flanking end-linker sequence TCGGTCACATGTGCATCCTCGATCTCA | | | This study | |  |  |
|  | **pGGC 44** | 1 | | GAGC | | CATC | | pUK21 | | Kan^R^ | yes | End-linker level 1 spanning position 6 till 7 based on pUC19 vector with additional integrated *BsaI/BpiI* restriction sites flanking end-linker sequence TCGGTCACATGTGCATCCTCGATCTCA | | | This study | |  |  |
|  | **pGGC 47** | 1 | | AGGA | | CATC | | pUK21 | | Kan^R^ | yes | End-linker level 1 spanning position 7 till 7 based on pUC19 vector with additional integrated *BsaI/BpiI* restriction sites flanking end-linker sequence TCGGTCACATGTGCATCCTCGATCTCA | | | This study | |  |  |
|  | **pGGC 46** | 2 | | GCCA | | CATC | | pBluescript II SK (+) | | Amp^R^ | yes | Level 2 empty entry vector based on pBluescript II SK (+) vector with additional integrated *BsaI* restriction sites flanking *lacZα* | | | This study | |  |  |
|  | **pGGC 208** | 2 | | GCCA | | CATC | | pSEVA 351 | | Amp^R^ | yes | Level 2 empty entry vector based on pSEVA 351 vector with additional integrated *lacZα* flanked by *BsaI* restriction sites | | | This study | |  |  |
|  | **pGGC 8** | 0 | | GATG | | AAAG | | pGGC 0 | | Amp^R^ | no | Level 0 *P_nrsB_* | | | This study | |  |  |
|  | **pGGC 9** | 0 | | GATG | | AAAG | | pGGC 0 | | Amp^R^ | no | Level 0 *P_rhaBAD_, rhaS* | | | This study | |  |  |
|  | **pGGC 10** | 0 | | GATG | | AAAG | | pGGC 0 | | Amp^R^ | no | Level 0 *P_psbA2_* | | | This study | |  |  |
|  | **pGGC 11** | 0 | | GATG | | AAAG | | pGGC 0 | | Amp^R^ | no | Level 0 Spec^R^ | | | This study | |  |  |
|  | **pGGC 12** | 0 | | GATG | | AAAG | | pGGC 0 | | Amp^R^ | no | Level 0 T*_psbC_* | | | This study | |  |  |
|  | **pGGC 13** | 0 | | GATG | | AAAG | | pGGC 0 | | Amp^R^ | no | Level 0 5’ hom. region | | | This study | |  |  |
|  | **pGGC 14** | 0 | | GATG | | AAAG | | pGGC 0 | | Amp^R^ | no | Level 0 3’ hom. region | | | This study | |  |  |
|  | **pGGC 15** | 0 | | GATG | | AAAG | | pGGC 0 | | Amp^R^ | no | Level 0 *Cnhox* operon | | | This study | |  |  |
|  | **pGGC 39** | 0 | | GATG | | AAAG | | pGGC 0 | | Amp^R^ | no | Level 0 pHG1_*hyp*X | | | This study | |  |  |
|  | **pGGC 57** | 0 | | GATG | | AAAG | | pGGC 0 | | Amp^R^ | no | Level 0 *P_J23119_*-*rhaS* | | | This study | |  |  |
|  | **pGGC 115** | 0 | | GATC | | AAAG | | pGGC 0 | | Amp^R^ | no | Level 0 *hyp* operon | | | This study | |  |  |
|  | **pGGC 16** | 1 | | GCCA | | GTTA | | pGGC 1 | | Kan^R^ | no | Level 1 position 1 5’ hom. region | | | This study | |  |  |
|  | **pGGC 17** | 1 | | GTTA | | CTAG | | pGGC 2 | | Kan^R^ | no | Level 1 position 2 *P_nrsB_* | | | This study | |  |  |
|  | **pGGC 18** | 1 | | GTTA | | CTAG | | pGGC 2 | | Kan^R^ | no | Level 1 position 2 *P_rhaBAD_* | | | This study | |  |  |
|  | **pGGC 19** | 1 | | GTTA | | CTAG | | pGGC 2 | | Kan^R^ | no | Level 1 position 2 *P_psbA2_* | | | This study | |  |  |
|  | **pGGC 20** | 1 | | CTAG | | CAGA | | pGGC 3 | | Kan^R^ | no | Level 1 position 3 *CnHox* operon | | | This study | |  |  |
|  | **pGGC 21** | 1 | | CAGA | | TGTG | | pGGC 4 | | Kan^R^ | no | Level 1 position 4 T*_psbC_* | | | This study | |  |  |
|  | **pGGC 22** | 1 | | TGTG | | GAGC | | pGGC 5 | | Kan^R^ | no | Level 1 position 5 Spec^R^ | | | This study | |  |  |
|  | **pGGC 23** | 1 | | GAGC | | AGGA | | pGGC 6 | | Kan^R^ | no | Level 1 position 6 3’ hom. region | | | This study | |  |  |
|  | **pGGC 82** | 1 | | CTAG | | CAGA | | pGGC 3 | | Kan^R^ | no | Level 1 position 3 *hypX* | | | This study | |  |  |
|  | **pGGC 85** | 1 | | GAGC | | AGGA | | pGGC 6 | | Kan^R^ | no | Level 1 position 6 *P_J23119_*-*rhaS* | | | This study | |  |  |
|  | **pGGC 86** | 1 | | TGTG | | GAGC | | pGGC 5 | | Kan^R^ | no | Level 1 position 5 *P_J23119_*-*rhaS* | | | This study | |  |  |
|  | **pGGC 116** | 1 | | CTAG | | CAGA | | pGGC 3 | | Kan^R^ | no | Level 1 position 3 *hyp* operon | | | This study | |  |  |
|  | **pGGC 209** | 2 | | GCCA | | CATC | | pGGC 208 | | Chl^R^ | no | Level 2 pGGC208 with pGGC 40; 17; 20; 21; 22 and 44 | | | This study | |  |  |
|  | **pGGC 212** | 2 | | GCCA | | CATC | | pGGC 46 | | Amp^R^ | no | Level 2 pGGC 46 with pGGC 16; 17; 20; 21; 22, 23 and 47 | | | This study | |  |  |
|  | **pGGC 243** | 2 | | GCCA | | CATC | | pGGC 208 | | Chl^R^ | no | Level 2 pGGC208 with pGGC 40; 17; 82; 21; 90 and 44 | | | This study | |  |  |
|  | **pGGC 244** | 2 | | GCCA | | CATC | | pGGC 208 | | Chl^R^ | no | Level 2 pGGC 208 with pGGC 40; 18; 82; 21; 85; 90 and 47 | | | This study | |  |  |
|  | **pGGC 271** | 2 | | GCCA | | CATC | | pGGC 208 | | Chl^R^ | no | Level 2 pGGC208 with pGGC 40; 17; 116; 21 and 43 | | | This study | |  |  |
|  | **pGGC 272** | 2 | | GCCA | | CATC | | pGGC 208 | | Chl^R^ | no | Level 2 pGGC 208 with pGGC 40; pGGC 18; 116; 21; 86 and 44 | | | This study | |  |  |
|  | **pGGC 273** | 2 | | GCCA | | CATC | | pGGC 208 | | Chl^R^ | no | Level 2 pGGC 208 with pGGC 40; pGGC 19; 116; 21; 86 and 43 | | | This study | |  |  |
|  |  |  | |  | |  | |  | |  |  |  | | |  | |  |  |
|  |  | |  | |  | |  | |  | |  | |  |  | |  |  |  |

**Table S4. Strains used in this study.**

| **Strain** | **characteristics** | **Reference** |
| --- | --- | --- |
| *E.coli* DH5α | F^–^ φ80*lac*ZΔM15 Δ(*lac*ZYA*arg*F)U169 *rec*A1  *end*A1 *hsd*R17(r_K_^–^, m_K_^+^) *pho*A *sup*E44 λ^–^*thi*-1 *gyr*A96 *rel*A1 | (Hanahan, 1983) |
| *Syn*_∆hox | *Synechocystis* strain in which the native *hox* operon was replaced by kan^R^ Antibiotic cassette. Integration of chl^R^ antibiotic cassette between *slr1597* and *sll1514* | (Appel *et al.*, 2020) |
| *Syn_Cn*SH^+^ | *hox* operon of *C. necator* was fused to the *psbA2* promoter; cassette was integrated into the original locus of the native *hox* genes from the WT (Δ*hox*::*PpsbA2hoxFUYHW*(PCC6803)) | (Lupacchini *et al.*, 2021) |
| *Syn_*P*_nrsB_Cn*SHg | *hox* operon as constructed in pGGC 212 was integrated into the original locus of the native *hox* genes from the WT (Δ*hox*::*PpsbA2hoxFUYHWI*(PCC6803)) | This study |
| *Syn_*P*_nrsB_Cn*SHp | The replicative plasmid containing the *hox* operon, pGGC 209 was introduced in *Syn*_∆*hox*. | This study |
| +p*P_nrsB_Cn*Hyp | *Syn_P_nrsB_Cn*SH*g* transformed with pGGC 271 pSEVA plasmid, harboring *Cn_hyp* synthetic operon fused to the *nrsB* promoter. | This study |
| +p*P_rhaBAD_Cn*Hyp | *Syn_P_nrsB_Cn*SH*g* transformed with pGGC 272 pSEVA plasmid harboring *Cn_hyp* synthetic operon fused to the *rhaBAD* promoter | This study |
| +p*P_psbA2_Cn*Hyp | *Syn_P_nrsB_Cn*SH*g* transformed with pGGC 273 pSEVA plasmid harboring *Cn_hyp* synthetic operon fused to the *psbA2* promoter | This study |
| +p*P_nrsB_Cn*HypX | *Syn_P_nrsB_Cn*SH*g* transformed with pGGC 243 pSEVA plasmid harboring the native sequence of *Cn_hypX* gene, fused to the *nrsB* promoter. | This study |
| +p*P_rhaBAD_Cn*HypX | *Syn_P_nrsB_Cn*SH*g* transformed with pGGC 244 pSEVA plasmid harboring the native sequence of *Cn_hypX* gene, fused to the *rhaBAD* promoter. | This study |

**Table S5. Gas concentrations during in vivo H_2_ consumption assay.** H_2_ and O_2_ concentrations in the cultures headspace during the 6-8 h incubation time. Data represent mean values and standard deviations of three biological replicates.

**
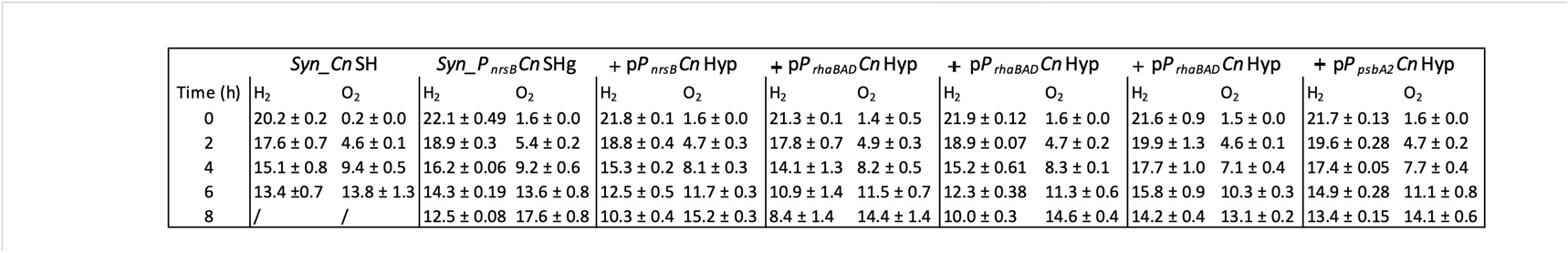
**

**Table S6. H_2_ uptake rate during H_2_ consumption assay.** Specific hydrogenase activity (U g_CDW_^-1^) measured for each strain during the 6-8 h incubation time

**
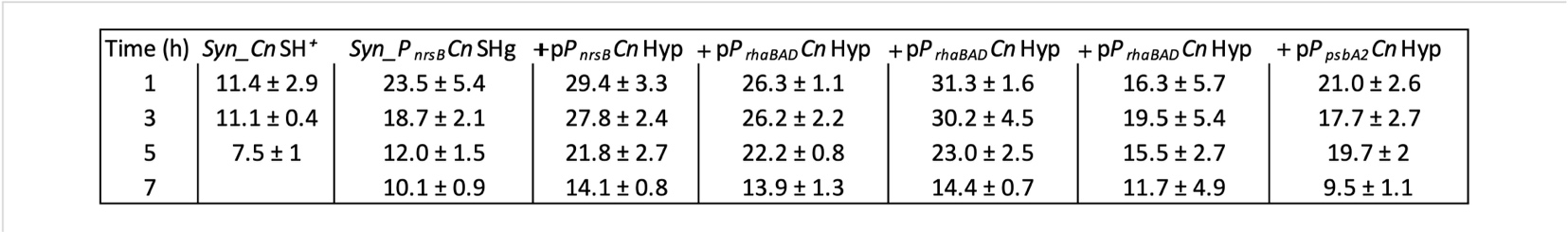
**

**References**

Appel J, Hueren V, Boehm M, and Gutekunst K. 2020. Cyanobacterial *in vivo* solar hydrogen production using a photosystem I–hydrogenase (PsaD-HoxYH) fusion complex, *Nature Energy*. Springer US, 5(6): 458–467.

Behle A, Saake P, Germann AT, Dienst D, and. Axmann IM. 2020. Comparative Dose-Response Analysis of Inducible Promoters in Cyanobacteria, *ACS Synthetic Biology*, 9(4): 843–855.

Hanahan, D. Studies on transformation of *Escherichia coli* with plasmids. *J Mol Biol* 166, 557-580 (1983).

Lonsdale TH, Lauterbach L, Honda Malca S, Nestl BM, Hauer B, and Lenz O. 2015. H_2_-driven biotransformation of n-octane to 1-octanol by a recombinant *Pseudomonas putida* strain co-synthesizing an O_2_-tolerant hydrogenase and a P450 monooxygenase, *Chemical Communications*. Royal Society of Chemistry, 51(90): 16173–16175.

Lupacchini S, Appel J, Stauder R, Bolay P, Klähn S, Lettau E, Adrian L, Lauterbach L, Bühler B, Schmid A, Toepel J*.* 2021. Rewiring cyanobacterial photosynthesis by the implementation of an oxygen-tolerant hydrogenase, *Metabolic Engineering*, 68:199–209.

Opel F, Itzenhäuser M. A, Wehner I, Lupacchini S, Lauterbach L, Lenz O, & Klähn S. 2023. Toward a synthetic hydrogen sensor in cyanobacteria: functional production of an oxygen-tolerant regulatory hydrogenase in *Synechocystis* sp. PCC 6803, 14:1122078.

Prentki P, and Krisch H. M. *In vitro* insertional mutagenesis with a selectable DNA fragment, *Gene*, 29, pp. 303–313 (1984).
